# Rapid Evolution of PARP Genes Suggests a Broad Role for ADP-Ribosylation in Host-Virus Conflicts

**DOI:** 10.1371/journal.pgen.1004403

**Published:** 2014-05-29

**Authors:** Matthew D. Daugherty, Janet M. Young, Julie A. Kerns, Harmit S. Malik

**Affiliations:** 1 Division of Basic Sciences, Fred Hutchinson Cancer Research Center, Seattle, Washington, United States of America; 2 Institute for Systems Biology, Seattle, Washington, United States of America; 3 Howard Hughes Medical Institute, Fred Hutchinson Cancer Research Center, Seattle, Washington, United States of America; Boston College, United States of America

## Abstract

Post-translational protein modifications such as phosphorylation and ubiquitinylation are common molecular targets of conflict between viruses and their hosts. However, the role of other post-translational modifications, such as ADP-ribosylation, in host-virus interactions is less well characterized. ADP-ribosylation is carried out by proteins encoded by the PARP (also called ARTD) gene family. The majority of the 17 human PARP genes are poorly characterized. However, one PARP protein, PARP13/ZAP, has broad antiviral activity and has evolved under positive (diversifying) selection in primates. Such evolution is typical of domains that are locked in antagonistic ‘arms races’ with viral factors. To identify additional PARP genes that may be involved in host-virus interactions, we performed evolutionary analyses on all primate PARP genes to search for signatures of rapid evolution. Contrary to expectations that most PARP genes are involved in ‘housekeeping’ functions, we found that nearly one-third of PARP genes are evolving under strong recurrent positive selection. We identified a >300 amino acid disordered region of *PARP4*, a component of cytoplasmic vault structures, to be rapidly evolving in several mammalian lineages, suggesting this region serves as an important host-pathogen specificity interface. We also found positive selection of *PARP9*, *14* and *15*, the only three human genes that contain both PARP domains and macrodomains. Macrodomains uniquely recognize, and in some cases can reverse, protein mono-ADP-ribosylation, and we observed strong signatures of recurrent positive selection throughout the macro-PARP macrodomains. Furthermore, *PARP14* and *PARP15* have undergone repeated rounds of gene birth and loss during vertebrate evolution, consistent with recurrent gene innovation. Together with previous studies that implicated several PARPs in immunity, as well as those that demonstrated a role for virally encoded macrodomains in host immune evasion, our evolutionary analyses suggest that addition, recognition and removal of ADP-ribosylation is a critical, underappreciated currency in host-virus conflicts.

## Author Summary

The outcome of viral infections is determined by the repertoire and specificity of the antiviral genes in a particular animal species. The identification of candidate immunity genes and mechanisms is a key step in describing this repertoire. Despite advances in genome sequencing, identification of antiviral genes has largely remained dependent on demonstration of their activity against candidate viruses. However, antiviral proteins that directly interact with viral targets or antagonists also bear signatures of recurrent evolutionary adaptation, which can be used to identify candidate antivirals. Here, we find that five out of seventeen genes that contain a domain that can catalyze the post-translational addition ADP-ribose to proteins bear such signatures of recurrent genetic innovation. In particular, we find that all the genes that encode both ADP-ribose addition (via PARP domains) as well as recognition and/or removal (via macro domains) activities have evolved under extremely strong diversifying selection in mammals. Furthermore, such genes have undergone multiple episodes of gene duplications and losses throughout mammalian evolution. Combined with the knowledge that some viruses also encode macro domains to counteract host immunity, our evolutionary analyses therefore implicate ADP-ribosylation as an underappreciated key step in antiviral defense in mammalian genomes.

## Introduction

Post-translational modifications (PTMs) of proteins regulate a wide variety of cellular processes, including several aspects of innate immunity against pathogens. As a result, pathogens have evolved mechanisms to block, reverse or usurp this machinery in order to successfully replicate within their hosts [1]. For example, numerous viruses subvert the dynamics of phosphorylation, employing kinases, substrate mimics and phosphatases to disrupt host signaling [1]. Likewise, addition and removal of acetyl groups by histone acetyltransferases (HATs) and deacetylases (HDACs) can have a dramatic effect on viruses such as HIV, herpesviruses, polyomaviruses and papillomaviruses. In response, several viral classes encode proteins to specifically disrupt host phosphorylation and acetylation [2]. Beyond small-molecule PTMs, conjugation and cleavage of ubiquitin and ubiquitin-like molecules has emerged as an important point of cellular regulation that several viruses target or subvert in order to replicate [3].

**Figure 1:**
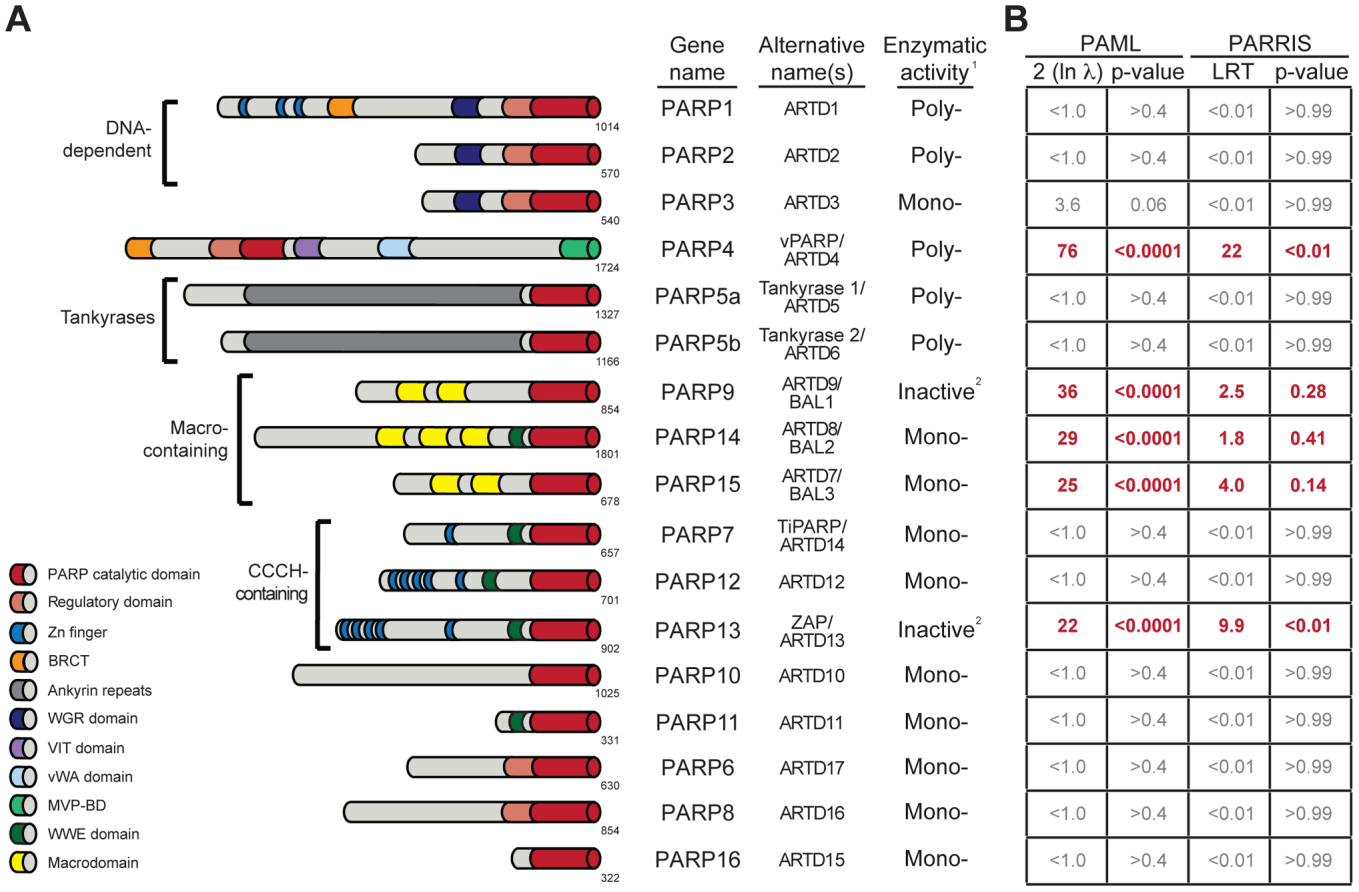
Several PARP genes are evolving under positive selection. (A) Schematic domain structures of human PARP proteins are shown (not to scale). Numbers to the bottom right of the protein schematic indicate the total length, in amino acids, of each protein. BRCT: BRCA1 C-terminus domain; VIT: vault inter-trypsin domain; vWA: von Willebrand factor type A; MVP-BD: major vault protein binding domain. ^1^PARPs are categorized as either poly-ADP-ribosyltransferases (Poly-), mono-ADP-ribosyltransferases (Mono-) or inactive based on presence of conserved motifs and, when available, data using enzymatic assays [4], [6]. ^2^PARP9 and PARP13 lack one or more catalytic residues conserved in all other PARPs and are therefore predicted to lack catalytic activity, although it is unknown whether they still bind ADP-ribose or ADP-ribosylated proteins [4]. (B) Results of maximum likelihood tests for positive selection. The five PARP genes showing strong signatures of positive selection are shown in red. Shown at left are values derived from PAML indicating twice the log difference between an evolutionary model that allows positive selection (M8) versus a model that disallows position selection (M8a). P-values indicate whether the model that allows positive selection better fits the data. PARRIS-derived LRT (likelihood ratio test) values also report the difference between the log-likelihood values for a model allowing positive selection versus a model that does not. Using seven orthologs in PARRIS only gave a statistically significant p-value (<0.01) for *PARP4* and *PARP13*, however analysis of additional sequences of *PARP9*, *14* and *15* found that these genes also met statistical significance (see Results). Additional analyses are reported in Figure S1.

In contrast, ADP-ribosylation is still poorly characterized for its role in innate immunity, despite being one of the first identified PTMs. Transfer of ADP-ribose (ADPr) from NAD^+^ (nicotinamide adenine dinucleotide) to proteins is catalyzed within eukaryotic cells by members of the PARP (poly-ADP-ribose polymerase), or ARTD (ADP-ribosyltransferase, diphtheria toxin-like) protein family (Figure 1A) [4], [5]. The best-studied PARPs, including the founding member PARP1, catalyze the formation of long, branched chains of ADP-ribose known as poly-ADP-ribose (PAR) [4], [6], [7], [8]. These PAR-forming enzymes perform critical housekeeping functions, such as nucleation of DNA-damage foci (PARP1 and 2) and proper chromosome segregation during mitosis (PARP5a) [7], [8]. In contrast to these well-described functions, most human PARP proteins are poorly understood, in part due to their lack of conservation in model organisms such as *C. elegans* and *D. melanogaster* [4], [9], [10]. In total, 17 genes in the human genome contain PARP domains, with each gene containing a variety of other functional domains that likely endow each PARP with their individual functions (Figure 1A) [4], [10]. Many of the poorly-characterized human PARP proteins are found in the cytoplasm [11] and are predicted to only catalyze addition of a single ADPr, rather than PAR, to proteins [4], [9], [10]. Several recent descriptions of PARP functions in cellular signaling, miRNA regulation and stress granule formation [12], [13], [14] suggest that many functions for cytoplasmic ADP-ribosylation, especially mono-ADP-ribosylation, likely remain uncharacterized. Moreover, the discovery that a subset of macrodomain containing proteins can, in addition to binding mono-ADP-ribosylated proteins, also remove mono-ADP-ribose from proteins [15], [16], sheds further light on the regulation and function of this dynamic PTM.

One function of ADP-ribosylation may be to regulate viral infectivity and pathogenesis, consistent with the role of other PTMs in immunity. For example, both vaccinia virus [17] and herpes simplex virus [18] require ADP-ribosylation activity for viral replication. Moreover, diverse RNA viruses, such as alphaviruses, hepatitis E virus, rubella virus and SARS coronavirus encode one or more macrodomains, potentially conferring the ability to specifically recognize, and possibly reverse, ADP-ribosylation upon these viruses [19]. Mutations in the macrodomain of Sindbis virus led to reduced virulence in mice [20]. Similarly, mutations in the SARS coronavirus macrodomain sensitized the virus to the antiviral effects of the signaling cytokine, interferon (IFN) [21]. As IFN functions as one of the primary mediators of the innate immune system against viruses [22], these results indicate that macrodomains, and therefore ADP-ribosylation, could be important viral regulators of host immunity. Moreover, host PARP genes can play a direct role in antiviral immunity. For example, overexpression of PARP13, also known as ZAP or ZC3HAV1 (Zinc-finger CCCH-type antiviral protein 1), is sufficient to restrict replication of several different families of viruses, including a retrovirus (murine leukemia virus [23]), filoviruses (Ebola and Marburg [24]), a togavirus (Sindbis [25]) and a hepadnavirus (Hepatitis B virus [26]). This antiviral activity is mediated through direct binding of viral RNA by PARP13, followed by recruitment of the exosome and specific degradation of viral RNA [27], [28], although more recently, additional signaling roles for PARP13 have been proposed [14], [29]. Beyond the well-described PARP13-mediated antiviral functions, PARP1, 7, 10 and 12 have been shown to play roles in repressing viral replication [30], [31], [32], [33], although the mechanisms of these antiviral actions are unknown. While these results indicate that there may be a role for individual PARPs in regulating viral infectivity or pathogenesis, there has been no cohesive model for how ADP-ribosylation may influence host-viral interactions.

We reasoned that if ADP-ribosylation is the focus of a host-virus conflict, we might see evolutionary signatures of positive (diversifying) selection acting on the specific host genes involved. Positive selection is a hallmark of host genes locked in genetic conflict with viruses that counter-evolve to evade the host antiviral defenses, and has been seen in both antiviral kinases and antiviral ubiquitin ligases [34]. Positive selection is characterized by the accumulation of amino acid-altering, nonsynonymous changes in the DNA at a rate that is greater than the accumulation of neutral, synonymous changes. When such protein changes are recurrently selected for (due to their adaptive advantage), the ratio of nonsynonymous to synonymous substitution rates exceeds one (dN/dS > 1, where dN is the nonsynonymous substitution rate and dS is the synonymous substitution rate). Such analyses can not only identify a gene that has evolved under positive selection but can also pinpoint domains and even individual codons within that gene located at the direct interface between host and viral factors [35], [36]. We previously analyzed primate *PARP13* orthologs to determine if the direct antiviral activity of PARP13 has led to a genetic conflict with viruses. Indeed, consistent with its antiviral function, we found a robust signature of positive selection in *PARP13* in primates [37]. Interestingly, despite the fact that the zinc-finger domains of PARP13 directly bind viral RNA [27], we found no signature of positive selection in these domains. Instead, we found sites of positive selection in the PARP catalytic domain, implying that this domain is a target for genetic conflict with viruses [37]. Although this domain in PARP13 appears to lack catalytic activity [4], we nevertheless found that its removal from PARP13 decreased the level of viral restriction [37], arguing that some function of the PARP domain remains intact. Thus, using an evolutionary signature of positive selection as a guide, we were able to identify a domain important for the antiviral activity of PARP13.

To address whether ADP-ribosylation plays a broad role in viral immunity, we wished to take a comprehensive evolutionary approach to look for evidence of rapid evolution in all of the human PARP genes. We reasoned that evolutionary signatures of recurrent adaptation, such as those previously observed in *PARP13*, might reveal other uncharacterized PARP proteins that are involved in host-virus interactions. We therefore screened all 17 human PARP genes and their primate orthologs for signatures of recurrent positive selection. Contrary to expectations that most PARP genes are involved in ‘housekeeping’ functions, we found that nearly one third of human PARP genes bore signatures of recurrent genetic conflicts. In addition to *PARP13*, our evolutionary screen revealed four other PARP genes that have evolved under very strong positive selection in primates: *PARP4*, *9*, *14* and *15*. Two of these genes (*PARP14* and *15*) have also undergone dramatic gene turnover (gain and loss) during vertebrate evolution, an additional hallmark of gene innovation also seen in innate immunity genes such as *APOBEC3* and *TRIM5* [38], [39]. Based on their rapid evolution, we hypothesize that these four additional PARP genes are involved in as-yet-undescribed host-virus conflicts. Importantly, we anticipate that the identification of these rapidly evolving PARP genes and domains will enable future experiments to elucidate the role ADP-ribosylation plays in viral replication and host immunity.

## Results

### At least five primate PARP genes have evolved under recurrent positive selection

Motivated by our hypothesis that ADP-ribosylation may be an important PTM in host-virus conflicts, and our prior use of positive selection analyses to identify an important antiviral domain in *PARP13*, we investigated whether any of the other 16 human PARP genes also show signatures of recurrent positive selection. We searched publicly available primate genome sequences and identified orthologs of all 17 human PARP family members from a minimum of four hominoids, two Old World monkeys and one New World monkey. We performed a series of maximum likelihood analyses to detect recurrent positive selection for each PARP gene alignment. These analyses determine whether a model allowing positive selection at a subset of amino acid residues is a statistically better fit to the sequence data than a model that does not allow for positive selection. Using PAML software [40], we found that five PARP genes showed highly statistically significant (p-values <0.0001) signatures of positive selection (Figure 1B). In addition to confirming our earlier findings on *PARP13*, we found that *PARP4* (also known as *vPARP*) and the three macrodomain-containing PARP genes (*PARP9/BAL1*, *PARP14/BAL2* and *PARP15/BAL3*) all show signatures of positive selection. We followed up our PAML analyses with the more conservative PARRIS software implemented in the HyPhy package [41], which takes into account recombination and variation in synonymous substitution rates across codons. Using PARRIS, we again found these five PARP genes to be clearly distinct from the remaining 12 as judged by likelihood ratio tests (LRT) allowing or disallowing positive selection (Figure 1B). While our limited screen of seven orthologs in PARRIS only gave a statistically significant p-value (<0.01) for *PARP4* and *PARP13*, analysis of additional sequences of *PARP9, PARP14* and *PARP15* met statistical significance (see below). Finally, we performed branch-site analyses [42] to look for episodic signatures of positive selection on all 17 primate PARPs. We found that only *PARP4*, *PARP9* and *PARP13* demonstrated statistically significant signatures of episodic positive selection (Figure S1). This initial screen might underestimate the total number of PARP genes evolving under positive selection, firstly because our search is restricted to the primate lineage (selection might have operated only in other mammalian lineages) and secondly because we use only seven orthologs. Although such small alignments may lack power to detect weak selection, previous simulation studies have shown that strong selection on a subset of residues can be detected using PAML even with rather limited species surveys [43]. Given the signatures of positive selection we observed for *PARP4*, *9*, *14* and *15* in this initial screen, we characterized these four genes in further detail, collecting additional orthologous sequences to examine which domains contain positively selected residues in order to create a model for how viral conflict may have driven their evolution.

### Highly localized signature of positive selection in *PARP4*

PARP4, also known as vPARP (vault PARP) is a catalytically active poly-ADP-ribosyltransferase that is a component of widely conserved, large cytoplasmic ribonucleoprotein structures known as “vaults”. Vaults are barrel-shaped particles composed of three proteins, MVP (major vault protein), PARP4, and TEP1 (telomerase associated protein), as well as vRNA (vault RNA) [44]. The function of vaults is unknown, but they have been implicated in drug resistance, cancer and immunity. In support of a role in immunity, MVP, the core structural component of the >10 mDa mass of vaults, is upregulated by IFN, and vaults are most highly expressed in immune cell types such as dendritic cells and macrophages [45].

**Figure 2:**
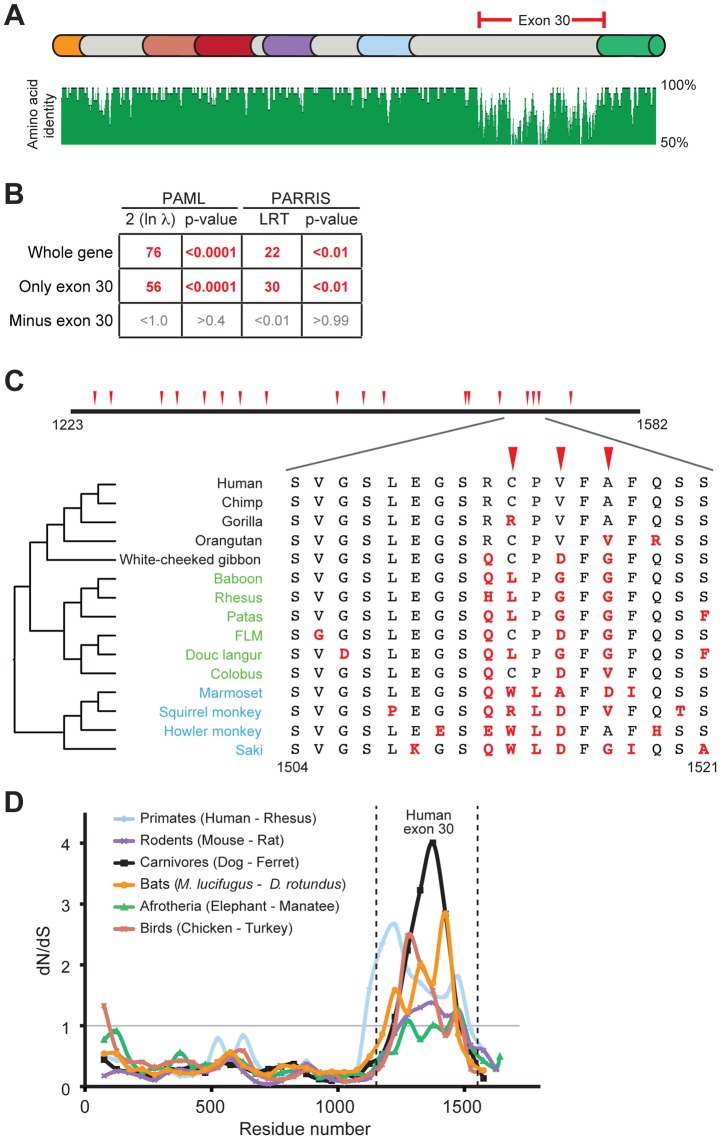
Positive selection in primate *PARP4* is localized to exon 30. (A) The domain structure of human PARP4 protein is shown with domain colors as in Figure 1A. The region of the protein corresponding to human exon 30 is bracketed in red. Also shown below the schematic is an amino acid identity plot calculated using seven publicly available primate PARP4 sequences. (B) Results of maximum likelihood tests for positive selection from seven publically available *PARP4* sequences using the entire gene, exon 30 alone or the entire gene except exon 30. Columns are as in Figure 1B. (C) An expanded view of exon 30 (residues 1223 to 1582 in human PARP4). Codons evolving under recurrent positive selection in an analysis of 15 primate species are marked with red triangles (posterior probabilities greater than 0.95) (see also Table S4). Below is a further expanded view of the alignment of residues 1504 to 1521 across all primates, with the phylogenetic tree shown to the left (Hominoids (black), Old World monkeys (green) and New World monkeys (blue)). Residues in red are changes from the human protein. (D) A sliding window dN/dS analysis of several pairs of vertebrate *PARP4* genes using a window size of 150 codons and a step size of 50 codons. The grey horizontal line marks a dN/dS value of 1, indicating neutral evolution. The vertical dashed lines outline the relative position of human exon 30. dN/dS values and confidence intervals from analyses of the largest exon of PARP4 from various vertebrate pairwise comparisons are shown in Table S3.

From the alignment of seven PARP4 orthologs, we noted a ∼360 amino acid region that was much more divergent than the rest of the protein (Figure 2A, Alignment S1). This protein segment is completely encoded by the largest exon of *PARP4* (exon 30 in humans). To illustrate the unusual selective pressures on exon 30, we performed a pairwise dN/dS comparison of human and rhesus *PARP4*s. We found that whereas the overall dN/dS ratio over *PARP4* is 0.63, the dN/dS ratio for exon 30 alone is 1.75 (>95% confidence for dN/dS > 1) (Table S3). This striking discrepancy between the evolution of exon 30 and the rest of the protein raised the possibility that this exon alone was responsible for the signature of positive selection in *PARP4*. We therefore repeated our positive selection analyses with exon 30 alone and found a robust signature of positive selection. In contrast, the remainder of *PARP4* showed no signature of positive selection upon removal of exon 30 (Figure 2B). Although we cannot formally rule out the possibility of weak selection acting outside exon 30 in PARP4, our analysis strongly suggests that exon 30 of *PARP4* has uniquely evolved under strong recurrent positive selection in primates. Because this evolutionary signature is isolated to a single exon, we next asked whether exon 30 is ever excluded from the *PARP4* transcript. We searched human and rhesus expressed sequence tag (EST) databases and found that all isoforms of *PARP4* include exon 30, suggesting that exon 30 is important for PARP4 function. Next, we searched the region encoded by exon 30 for sequence or structural homology to other protein domains. Surprisingly, secondary structure prediction software (JPRED [46]) indicated that the region encoded by exon 30 in human *PARP4* is almost entirely disordered. Taken together, we conclude that *PARP4* has evolved under recurrent positive selection in primates, but that positive selection is focused on the disordered region encoded by exon 30 alone.

We explored the signature of adaptive evolution in exon 30 of *PARP4* in more detail by sequencing exon 30 from genomic DNA from additional primates (Table S1). Analysis of a total of 15 primate *PARP4* exon 30 sequences confirmed our initial screening results that this region has evolved under positive selection (PAML p-value <0.0001, PARRIS p-value <0.01) (Figure S2A and Alignment S1). These analyses also identified several codons within exon 30 that display dramatic signatures of recurrent positive selection (Table S4). For instance, despite being in close proximity in the primary sequence to codons that are strictly conserved across primates, codon 1517 has undergone at least six amino acid changes during approximately 45 million years of simian primate evolution, with a calculated dN/dS ratio >3 (Figure 2C).

We also found that this pattern of rapid evolution in exon 30 extends to other vertebrate lineages. Despite high conservation in the rest of the PARP4 protein, the sequence and length of the largest exon (corresponding to human exon 30) in PARP4 is highly variable among vertebrates. Consistent with our results in primates, all closely related pairs of vertebrate *PARP4* orthologs analyzed demonstrated a signature of purifying selection throughout much of *PARP4* contrasting with evidence for positive selection in the region corresponding to exon 30 of human *PARP4* (Figure 2D and Table S3). To gain further insight into *PARP4* evolution outside of primates, we asked whether other mammalian lineages show evidence for recurrent positive selection as we observed in primates. To do this, we took advantage of publicly available bat genome sequences, which, like primates, are divergent enough to provide sufficient evolutionary divergence, but not so divergent that the rate of synonymous mutation (dS) is saturated. Using sequences from 10 bat species (Alignment S2), we again found that *PARP4* has evolved under recurrent positive selection in its largest exon (PAML p-value <0.0001, PARRIS p-value <0.01) (Figure S2B-C). PAML identified six positively sites with high confidence (Figure S2B-C, Table S5). Although there is no overlap between positively selected sites identified in primates and bats, we found nine residues to be absolutely conserved across all 25 primate and bat species we analyzed (Figure S2B-C), suggesting substantial constraint even within this rapidly evolving disordered protein domain. Combined, these broader phylogenetic analyses indicate that a single PARP4 region has been subject to positive selection throughout mammalian and bird evolution, suggestive of an ancient conflict with intracellular pathogens.

### Distinct patterns of positive selection in macrodomains of macro-PARP genes

Our evolutionary screen also revealed strong signatures of positive selection in *PARP9*, *PARP14* and *PARP15*. Strikingly, these three genes encode the only three human proteins that contain both a PARP catalytic domain and macrodomains, and are the only human genes to encode more than one macrodomain. The macrodomain is unique among protein domains in its ability to recognize mono-ADP-ribosylated proteins [47]. Furthermore, some macrodomains have recently been shown to catalyze the removal of mono-ADPr [15], [16]. Although the molecular functions of macro-PARPs are unclear, the presence of both PARP domains and macrodomains may conceivably allow them to both add and specifically recognize and/or reverse protein ADP-ribosylation. This, combined with the presence of macrodomains in viruses, prompted us to explore in more depth the evolution of other human macrodomain-containing proteins and ADP-ribosylhydrolases. Apart from the macro-PARPs, we found no evidence for positive selection in any other human gene encoding a macrodomain or ADP-ribosylhydrolase (Figure S3), suggesting that the combination of the macro- and PARP domains is important for their rapid evolution and, consequently, for their putative antiviral roles.

**Figure 3:**
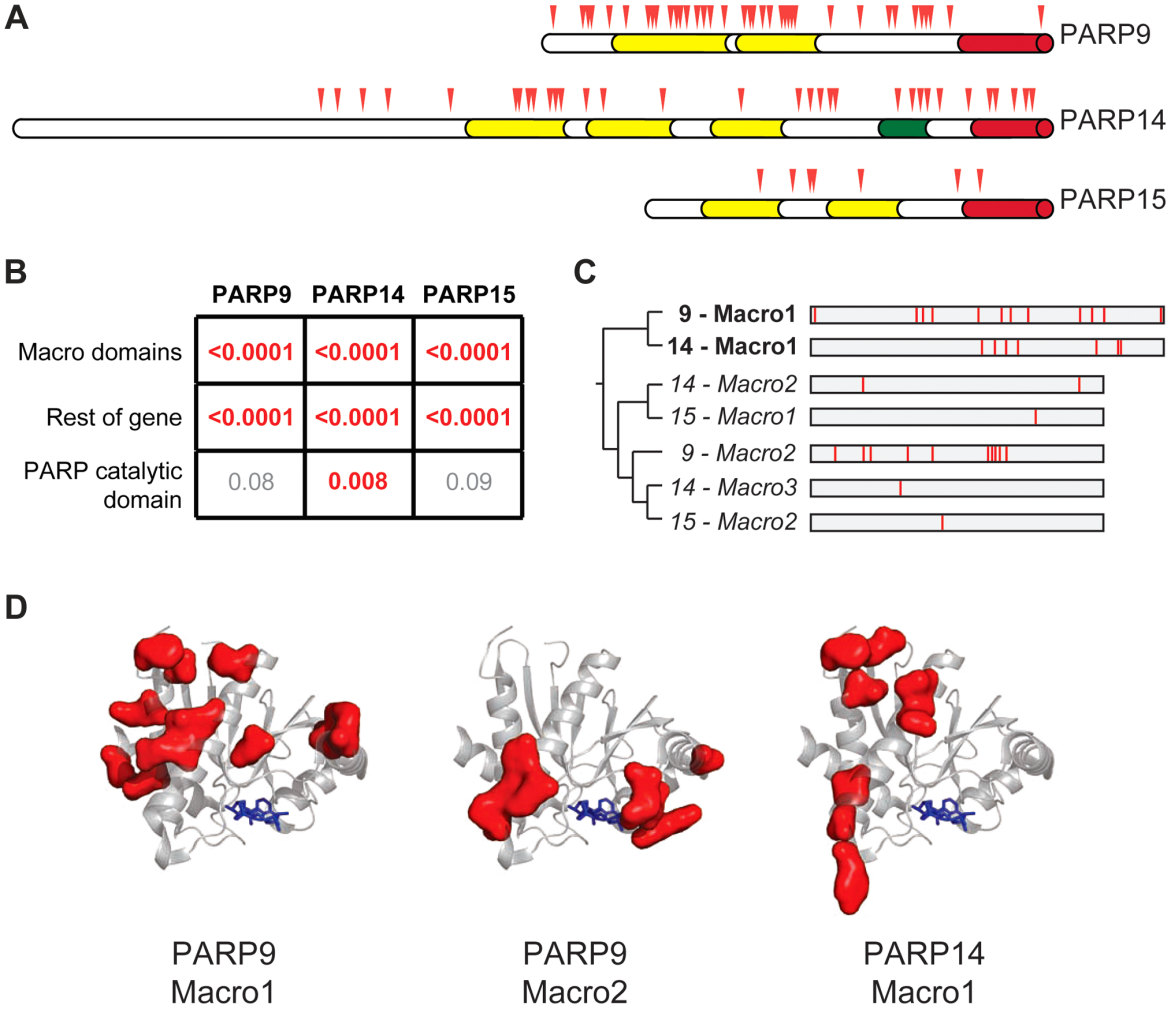
Widespread distribution of positive selection in macro-PARP genes. (A) The domain structure of each macro-PARP gene is shown with domain colors as in Figure 1A. Codons evolving under recurrent positive selection are marked with red triangles as in Figure 2 (see also Tables S6-S8). (B) P–values derived from PAML tests of positive selection on all sequenced macro-PARP orthologs using either macrodomains alone, the entire gene minus the macrodomains or just the catalytic domain. Values in red indicate strong evidence of positive selection, while those in grey indicate a lack of statistically significant evidence for positive selection. (C) Phylogenetic tree of individual macrodomains from macro-PARPs, rooted using human MacroD2 as an outgroup. Macrodomains in italics lack a C-terminal extension found in most macrodomains and lack one or more of the putative catalytic motifs required for ADP-ribosylhydrolase activity [16] (Figure S5). This suggests these macrodomains may be able to recognize, but are unlikely to catalyze removal of, ADP-ribosylation. To the right is a schematic of each macrodomain with positively selected residues indicated in red (posterior probabilities greater than 0.95) (see also Alignment S3). (D) Location of positively selected sites in PARP macrodomains mapped on to the structure of the first macrodomain from PARP14 in complex with ADP-ribose (PDB code: 3Q6Z)[48]. ADP-ribose is shown as blue sticks. Residues shown as red surfaces are those that have evolved under positive selection in the indicated macrodomain.

In order to further pinpoint which domains and codons in the macro-PARP genes have evolved under positive selection, we sequenced additional macro-PARP orthologs from a diverse panel of primates. Combining these with publically available sequences, we aligned and analyzed 15 or more orthologs for each macro-PARP gene (Table S1, Figure S4). Based on these expanded alignments, we confirmed the results of our initial screen; all macro-PARP genes have evolved under positive selection in simian primates (PAML p-value <0.0001, PARRIS p-value <0.01). In contrast to the recurrent positive selection on only a single exon of *PARP4*, we found that positively selected sites were broadly distributed throughout the macro-PARP genes (Figure 3A). For all three macro-PARPs, we observed strong evidence of positive selection acting on the macrodomains. However, removal of the macrodomain-containing segments did not result in a loss of positive selection signatures, indicating that both macrodomains as well as other domains have evolved under positive selection (Figure 3B). For instance, we found significant evidence for positive selection in the PARP domain of *PARP14* (Figure 3B), similar to *PARP13* [37]. In contrast, our analyses did not reveal evidence of positive selection acting on the PARP domains of *PARP9* and *PARP15* (Figure 3B), although it is possible that sequencing of additional orthologs might reveal more subtle signatures of selection. Thus, we conclude that macro-PARPs are evolving very rapidly, including substantial positive selection in the macrodomains of all three macro-PARPs.

Our finding that macrodomains encoded by macro-PARP genes have evolved under positive selection motivated us to investigate whether equivalent residues were rapidly evolving in each macrodomain. Such a conserved pattern could suggest that related genetic conflicts (for example, similar viral pathogens) drove their evolution. Instead, we observed that a different set of residues is rapidly evolving in each macro domain at a primary sequence level (Figure 3C, Tables S6-S8 and Alignment S3). While equivalent amino acids are not evolving in all macro-PARPs, it is possible that positive selection has acted on a single three-dimensional protein surface. We therefore modeled the positively selected residues from PARP9 and PARP14 macrodomains onto a structure that has been determined for the first macrodomain of PARP14 [48]. We found that positively selected residues map to a single surface of each macrodomain, but that each macrodomain shows positive selection on a distinct surface (Figure 3D). As each positively-selected surface is distinct relative to the site of ADP-ribose binding, these results suggest that ADP-ribose binding is not being altered or optimized by positive selection of the macrodomains. Instead, our findings suggest that each macrodomain has engaged in its own evolutionary arms race with as-yet-unidentified pathogen factors (see Discussion).

### Frequent gene turnover of macro-PARP genes *PARP14* and *PARP15*

Because most antiviral genes do not serve essential housekeeping functions, they can be lost during periods when selective pressures are relieved, for example during periods when fewer relevant viral pathogens are prevalent in the population. In contrast, selection to increase the breadth of antiviral specificities could also lead to increase in gene copy number [34]. As a result of these repeated rounds of innovations, many organisms undergo dramatic changes in their antiviral gene repertoires over evolutionary timeframes, as has been observed with *APOBEC* and *TRIM* genes in mammals [38], [39]. In our initial evolutionary screen, we had observed that most of *PARP15* is missing from the white-cheeked gibbon genome. Coupled with previous findings of *PARP15* absence in the mouse genome [10], we therefore investigated PARP genes in general, with an emphasis on the macro-PARP genes, for signatures of rapid gene turnover.

**Figure 4:**
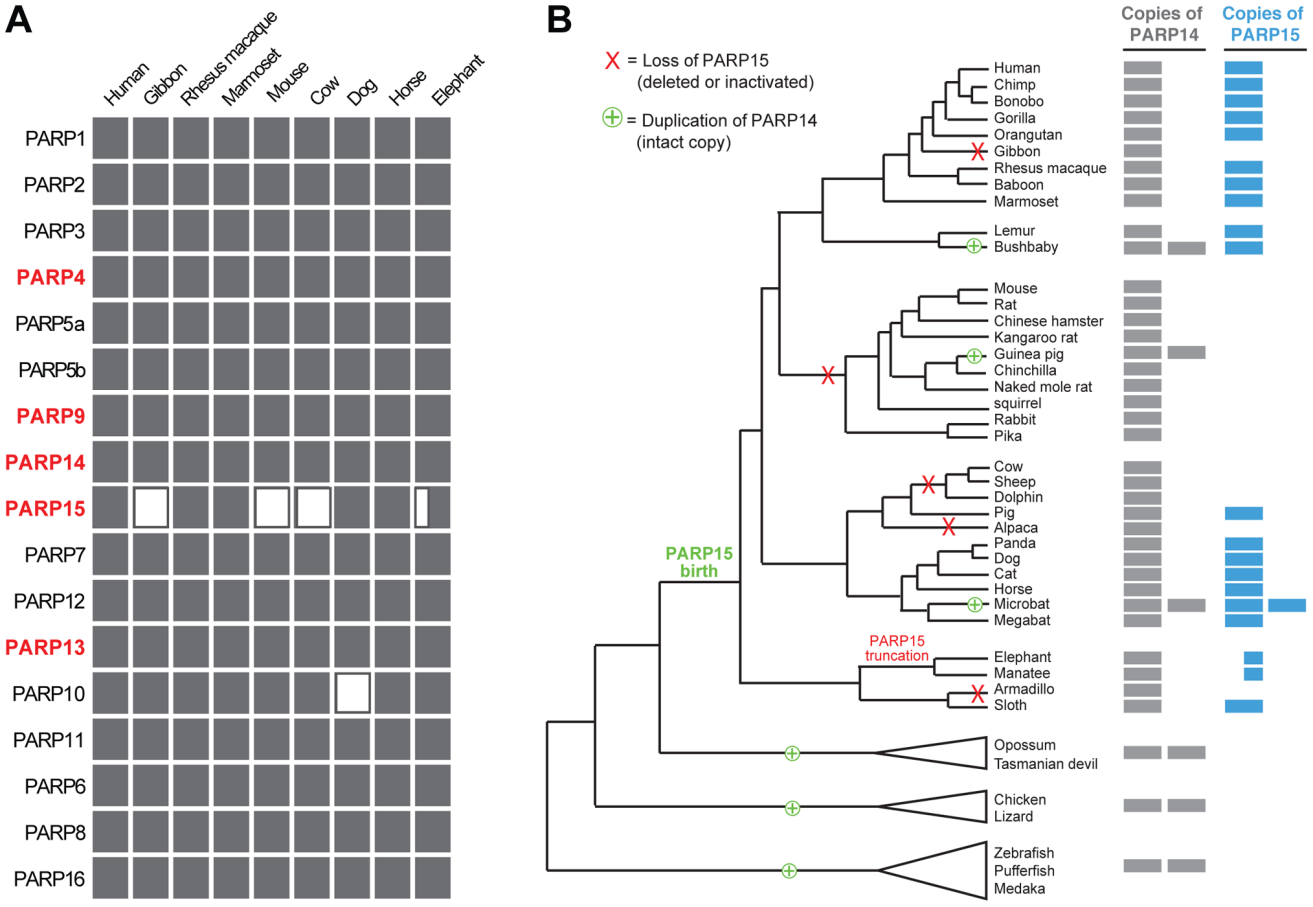
Recurrent gain and loss of *PARP14* and *PARP15*. (A) Intact PARP genes were identified in representative (high quality assembly) mammalian genomes. Filled rectangles indicate the presence of an intact gene, open rectangles indicate a gene loss. The half-filled rectangle indicates a large N-terminal truncation of *PARP15* in the elephant genome. (B) Intact *PARP14* and *15* homologs were compiled from publicly available vertebrate genomes (see Figure S6). A phylogenetic tree of queried species is shown with *PARP14* duplications (including the birth of *PARP15*) and losses of *PARP15* indicated. To the right is an indication of the number of intact *PARP14* and *PARP15* homologs in each genome, as indicated by the filled rectangles.

From our investigation of all seventeen *PARP* genes across a wide range of vertebrates, we found that *PARP15* is unique in its pattern of recurrent loss (Figure 4A). In contrast, other PARP genes are present in all genomes we examined, with the exception of *PARP10*, which has been lost in the carnivore lineage. To explore the dynamics of *PARP15* birth and loss, we conducted a more in-depth survey of *PARP15* genes in vertebrate genomes (Figure S5). We found that *PARP15* was born early in mammalian evolution via a partial duplication of *PARP14*, consisting of the second and third macrodomains and the PARP domain. We found that *PARP15* has been independently lost via deletion or inactivating mutations in five different mammalian lineages; PARP15 is therefore absent from gibbons, all glires (rodents and lagomorphs), the cow/sheep/dolphin clade, alpaca/camel, and armadillo (Figure 4B). Elephant and manatee have a conserved but shorter form of PARP15, missing the first of the two macrodomains. In contrast to these losses in *PARP15*, we identified several *PARP14* duplications that occurred both within and outside the lineage that contains *PARP15*. For instance, although fish and birds lack *PARP15* orthologs, many fish and bird genomes have one or more additional copies of *PARP14* that could possibly serve *PARP15*-analogous functions. Guinea pig and bushbaby each appear to have at least one extra intact copy of *PARP14*, with the caveat that in each case a single exon is within a genome assembly gap. The microbat (*Myotis lucifigus*) genome contains at least eight *PARP14/15* genes, of which at least two copies are intact (two additional genes are incomplete in the assembly but are uninterrupted in available sequence by stop codons or frameshifts, suggesting they are also intact) (Figure 4B). Moreover, pairwise comparisons of duplicated PARP14 genes in microbat and bushbaby suggest that these paralogs may have regions that have rapidly diverged under positive selection (Figure S7), although additional sequences will be required to strengthen such a conclusion. Coupled with our findings that both *PARP14* and *PARP15* are evolving under positive selection in primates (Figure 3), the gene turnover we describe for *PARP14* and *PARP15* supports the idea that these genes have been selected for functional innovation, perhaps in response to a recurrent genetic conflict with pathogens.

## Discussion

Post-translational protein modifications are a common regulatory mechanism for modulating protein activity, stability and localization. As such, numerous viruses manipulate host PTM machinery to regulate their own replication or evade host antiviral immunity. Research aimed at understanding these viral strategies has provided critical insight into the host processes mediated by PTMs, including tyrosine phosphorylation and regulation of histone acetylation [1], [2]. Inspired by the fact that signatures of positive selection can be used to highlight important genes and PTMs in host-virus conflicts, we performed an evolutionary screen on all of the primate PARP genes to ask if ADP-ribosylation is an important player in host-virus dynamics. Contrary to what would be expected of a PTM that is solely dedicated to housekeeping functions, we found strong evidence for rapid evolution in five of seventeen primate PARP genes, suggesting a broad involvement for PARPs, and ADP-ribosylation, in genetic conflicts. Moreover, we observed evolutionary signatures that suggested an ancient history of conflict for these PARP genes. For example, we see positive selection on *PARP4* in diverse mammalian clades and recurrent gain and loss of *PARP14* and *PARP15* across vertebrates. Our findings suggest that *PARP4*, *9*, *13*, *14* and *15* are each locked in a genetic conflict, likely with one or more pathogenic agents.

Our data do not exclude the possibility that other genetic conflicts, perhaps in addition to viral conflicts, drove PARP positive selection. Indeed, the first discovery of manipulation of host processes by ADP-ribosylation emerged from the study of bacterial toxins (e.g., diphtheria, cholera toxins) [49], leaving open the possibility that bacterial or eukaryotic pathogens drove the evolution of PARP genes. However, we hypothesize that viruses may be significant or even the primary pathogens in these evolutionary arms races for several reasons. First, numerous viruses replicate poorly when ADP-ribosylation is inhibited, including viruses that replicate in the nucleus (HSV) [18] and cytoplasm (vaccinia) [17]. Second, several families of mammalian RNA viruses, including corona- and togaviruses, have non-structural proteins that contain macrodomains. In both corona- and togaviruses, disruption of viral macrodomains has been shown to reduce virulence [20], [21], and in the case of coronaviruses, this reduced virulence is due to increased sensitivity to the antiviral activity of interferon (IFN) [21]. This suggests a simple model in which the macrodomains (at least in coronaviruses) are required to counteract some IFN-stimulated host gene product. Although the identity of that IFN-stimulated factor is unknown, we note that several of the rapidly evolving PARP genes we identify here, including *PARP9*, *PARP13* and *PARP14*, are upregulated by IFN [50], [51]. Furthermore, overexpression of PARP9, independent of IFN, is sufficient to upregulate several known antiviral effectors [50]. Finally, overexpression of several PARP genes has been shown to inhibit replication of viruses, the most well-described example being *PARP13*. Taken together, we favor a model in which PARP gene evolution has been driven primarily by genetic conflicts with viruses.

The patterns of evolution of the PARP genes allow us to make several inferences about the role of these proteins in genetic conflicts. First, the fact that we observe a robust evolutionary signature of positive selection in *PARP4*, *9*, *13*, *14* and *15* argues strongly that these genes are important for organismal fitness. Similar to strong evolutionary conservation, signatures of positive selection indicate that fixation of a particular allele, in this case, a novel allele, results in a strong enhancement of fitness. While rapid evolution may seem antithetical to functional constraint, in fact positive selection is a common hallmark of critical host immunity genes [34]. Thus, we infer that the functions of the rapidly evolving PARP genes we have identified are important for fitness in the face of rapidly-evolving pathogens. Second, we also find that *PARP14* and *PARP15* show recurrent gene duplication and loss. This form of genetic innovation is another common hallmark of immunity genes. Gene losses occur during periods of relaxed selection due to non-exposure or extinction of relevant pathogen(s), whereas gene duplications often provide additional genetic substrates for diversifying selection to increase anti-pathogen repertoires [34], [52]. While other PARP proteins, such as PARP1, PARP7, PARP10 and PARP12 [30], [31], [32], [33], have been identified as having antiviral functions, our initial screen suggests they have not been subject to strong recurrent antagonistic evolution with viral factors in primates, perhaps because their encoded proteins do not directly interact with virus-encoded factors.

Instead, our analyses lead to our novel hypotheses that PARP4, PARP9, PARP14 and PARP15, as well as the molecular complexes they reside in, possess antiviral activity. For instance, PARP4 is a component of large cytoplasmic structures known as vaults, whose functions are poorly understood. Although vaults are extremely ancient, dating back to the origin of eukaryotes, they have been lost in multiple lineages [9], suggesting that they are not universally necessary to perform an essential, housekeeping function. Instead, there are several tantalizing pieces of evidence that vaults may be involved in immunity. These include an increased number of vaults in immune cell types, IFN-upregulation of MVP, the major component of vaults, and upregulation of noncoding vault RNAs (vRNAs) on infection with pathogens such as Epstein-Barr virus [45]. PARP4 itself is present at ∼10 molecules per vault, but its functional role there is unknown [44]. However, our observation that the positively selected residues we find in PARP4 are localized to a single disordered region in PARP4 suggests a model for its role in vault-mediated immunity. Such a localized pattern of positively selected sites is reminiscent of two well-characterized rapidly evolving antiviral factors, TRIM5a and MxA, shown to be on the 'offensive' (*i.e.* directly binding to viral proteins) side of the host-virus conflict [34]. TRIM5a and MxA both use their rapidly evolving regions, also in the context of multimeric complexes, to directly recognize and target viral proteins, lentiviral capsids in the case of TRIM5a and orthomyxovirus nucleoproteins in the case of MxA [35], [36]. Thus, we infer that the positively selected region of PARP4 (exon 30 in humans) has evolved to maintain recognition of a factor encoded by pathogens that can infect many diverse mammalian lineages, or is a common means to counteract independent unrelated pathogens. This interaction may be used to directly ADP-ribosylate viral components, which could affect their activity and impede infection. Alternatively, independent of ADP-ribosylation, PARP4 interaction may recruit viral proteins to the vault structures within virally infected cells, wherein the vault proteins might sequester viral proteins and thereby impede their infectivity.

Likewise, our data highlight macrodomains as likely focal points of host-virus conflicts. However, in contrast to PARP4, which we hypothesize is on the ‘offense’ *i.e.*, specifically targeting viral proteins, the widely distributed pattern of positively selected sites within the macro-PARPs is reminiscent of other host immunity factors that are evolving on the ‘defensive’ side (i.e., directly targeted by viral antagonists) of evolutionary arms races [34]. In these cases, the host factor is performing a general antiviral function, such as shutting down host mRNA translation in the case of the antiviral protein PKR (Protein Kinase R), or transducing an antiviral transcriptional program as in the case of the antiviral protein MAVS (Mitochondrial Antiviral Signaling). As a result of their broad action, PKR and MAVS are antagonized by an array of proteins from diverse viral lineages that interact with different regions on the proteins [53], [54], [55], [56], [57]. The widely distributed pattern of positive selection in PKR [58] and MAVS [59] likely reflects this ‘defense’ against (or escape from) multiple antagonists. We postulate that the dispersed pattern of selection we observe in *PARP9*, *14* and *15* similarly reflects selection to escape recognition by a variety of distinct viral antagonists of a hypothesized PARP-mediated antiviral response (see below), rather than selection to maintain or establish recognition of viral target proteins.

**Figure 5:**
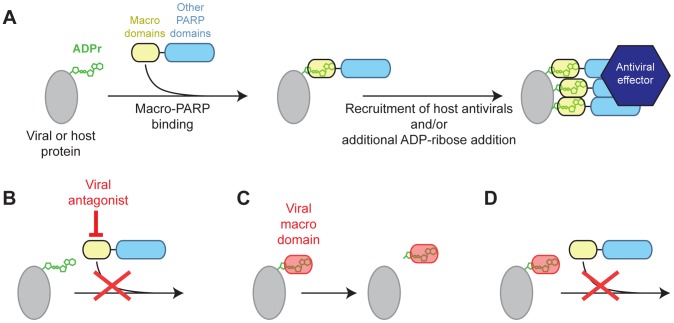
Model for genetic conflict involving PARP macrodomains. (A) Model for macro-PARP function. ADP-ribosylated host or viral proteins may be a signal for recruitment of PARP9, 14 or 15, which could facilitate additional recruitment of antiviral effectors, and amplify the initial ADPr signal. (B–D) Three models for how viruses may antagonize macro-PARP function. Viruses lacking their own macrodomains may use other proteins to directly antagonize macro-PARP proteins (B), driving recurrent positive selection in macro-PARP genes to escape antagonism. Macrodomains encoded by viruses (e.g. corona- and togaviruses) may catalyze the removal of ADPr (C) or compete with macro-PARPs for binding to ADPr (D) in order to antagonize host ADPr-mediated signaling.

This ‘defense’ model to explain macro-PARP evolution, combined with the importance of virally encoded macrodomains for the fitness of several RNA viruses, allows us to generate a mechanistic hypothesis for the conflict that may have driven the rapid evolution of macro-PARP genes. In our model, ADP-ribosylation functions as a post-translational modification of either host or viral factors (Figure 5A). We posit that macro-PARP proteins are recruited to sites of ADP-ribosylation by their macrodomains, most of which are predicted to be able to recognize, but not remove ADP-ribose (Figure S5), to exert either direct antiviral functions or recruit other antiviral factors. Recruitment of catalytically active macro-PARP genes could also facilitate further ADP-ribosylation of target proteins, allowing macro-PARP proteins to rapidly ‘amplify’ an initial signal of ADPr. Such a model of amplification by recruitment and additional ADP-ribosylation by PARP proteins has been seen at sites of DNA damage, where PARP1 activation by ADPr leads to increased ADP-ribosylation [7], [8]. We hypothesize that some viruses have overcome this macro-PARP-mediated antiviral response by direct antagonism of macro-PARP proteins (Figure 5B). Such antagonism could drive the rapid evolution we see in several regions of the macro-PARP genes, including but not limited to the macrodomains. However, other viruses, such as togaviruses and coronaviruses, have evolved their own macrodomains to either cleave ADP-ribose (Figure 5C) or compete with macro-PARP proteins (Figure 5D), in order to overcome the effects of the macro-PARP proteins. Although speculative, our model provides testable hypotheses about the genetic and physical interactions between PARP macrodomains, viral macrodomains, and ADP-ribose, and their consequences in terms of determining outcomes of viral infections in cells.

The exact antiviral consequences of macro-PARP action, and their cellular context, still remain unclear. However, recent studies on macro-PARPs implicate two candidate cellular processes. First, several PARP proteins (PARPs 5a, PARP13, PARP14 and PARP15) have been shown to be important for nucleation of stress granules in the cytoplasm, with ADP-ribosylation modulating miRNA activities [14], [29]. This suggests that ADP-ribose in stress granules, or the miRNA functions that are altered by ADP-ribosylation, are targets for arms races with viruses. Stress granules have been shown to have antiviral properties stemming from mRNA sequestration, degradation and translational repression [60]. In contrast, several viruses localize to stress granules and employ them for replication [60]. Our model suggests that stress granule-associated PARP genes may be evolving to either combat the hijacking of stress granules or miRNA by viruses, or as direct mediators of the antiviral functions of stress granules or miRNAs. Alternatively, macro-PARPs may act at the level of gene expression, where ADP-ribosylation and macro-PARPs may influence transcription regulatory complexes. Indeed, after the initial discovery that PARP9 was highly expressed in aggressive B-cell lymphomas [61], PARP9 and 14 were shown to regulate expression of several immunity-related genes [50], [62]. Thus, one possible explanation for positive selection in the macro-PARP genes is that viral antagonists target them to prevent transcription of antiviral genes. Such viral antagonism could not only inform us of the role for macro-PARPs in the cells, but could also be used as a guide to devise useful interventions for treatment of the aggressive lymphomas that are associated with high PARP9 expression. Whether macro-PARPs are operating in stress granules or for antiviral transcription, or in both processes, our model suggest that host macrodomains, and ADP-ribosylation, play a critical role in formation of antiviral complexes, whereas viruses actively target these complexes for antagonism.

To summarize, our evolutionary findings of recurrent positive selection of five PARP genes and gene turnover in two of those genes, together with previous observations by others (inhibition of viral replication by some PARP genes, viral modulation by chemical inhibitors of PARP activity, pathogenicity dependent on virally-encoded macrodomains in diverse RNA viruses) argue that ADP-ribosylation is a fundamental determinant of host-virus conflicts. Our results raise compelling hypotheses for the function of rapidly evolving PARP genes in these conflicts, and highlight the insights that can be gained from evolutionary analyses of previously poorly characterized genes.

## Materials and Methods

### Publically available primate PARP sequences

Publically available genome assemblies from human (*Homo sapiens*), chimpanzee (*Pan troglodytes*), orangutan (*Pongo abelii*), white-cheeked gibbon (*Nomascus leucogenys*), rhesus macaque (*Macaca mulatta*), baboon (*Papio anubis*) and marmoset (*Callithrix jacchus*) were queried for PARP genes. The *PARP12* gene is in a poorly assembled region of the orangutan genome and was therefore incomplete. The *PARP15* gene is almost entirely deleted from the white-cheeked gibbon genome; exon 4 is still present but contains a stop codon. We therefore used *PARP12* and *PARP15* sequences from the publically available gorilla (*Gorilla gorilla*) genome assembly to ensure that seven primate sequences were used in all analyses.

### Sequencing of primate PARP genes

*PARP4* exon 30 sequences were amplified from DNA isolated from cell lines obtained from Coriell Cell Repositories (Camden, NJ), the FrozenZoo (San Diego, CA), ATCC (Manassas, VA) and the Tulane National Primate Research Center (Covington, LA) (Table S1). Sequences were amplified by PCR using Phusion (New England Biolabs) polymerase using primers that anneal to the introns around exon 30 (primer sequences in Table S2). *PARP9*, *14* and *15* sequences were amplified from RNA isolated from cell lines (Table S1). Sequences were amplified by one step reverse-transcription PCR using SuperScript III One-Step RT-PCR with Platinum Taq (Invitrogen) to produce complementary DNA (cDNA) using the primers listed in Table S2. Repeated attempts to amplify *PARP15* from Siamang gibbon failed, supporting the conclusion that gibbons have lost *PARP15*. cDNAs were directly sequenced using internal primers by Sanger sequencing. Sequences from gorilla and squirrel monkey (*Saimiri boliviensis*) were obtained from publically available genome assemblies.

*PARP4* exon 30 sequences from primates have been deposited to Genbank under accession numbers KJ699095-KJ699100. *PARP9*, *14* and *15* mRNA sequences have been deposited to Genbank under accession numbers KJ697725-KJ697749.

### Tests for positive selection

PARP sequences were aligned in Geneious [63] and alignments were edited to remove gaps and ambiguities. Maximum likelihood (ML) tests were performed with codeml in the PAML software suite [40]. Aligned sequences were subjected to ML tests using NS sites models disallowing (M8a) or allowing (M8) positive selection. The p-value reported is the result of a chi-squared test on twice the difference of the likelihood values between the two models using one degree of freedom. All analyses were consistent when performed with varying models of codon frequency (F61 and F3×4) and varied starting omega values (0.4 and 1.5). Residues with recurrent signatures of positive selection with a posterior probability greater than 0.95 were identified using a Bayes Empirical Bayes (BEB) analysis in PAML and the F3×4 codon frequency model. A second set of maximum likelihood tests was performed using PARRIS in the HyPhy software suite [41], which also compares models disallowing or allowing positive selection. We report twice the difference in the log likelihood values (LRT), and a p-value based on that difference. Signatures of episodic positive selection were calculated in two ways. Overall dN/dS ratios for each branch of the phylogenetic tree were calculated using the free ratio model in PAML. A branch-site test (Branch-site REL [42]) for statistically significant signatures of episodic positive selection was performed using the HyPhy software suite [41].

K-estimator [64] was used for all pairwise sequence analyses of dN/dS ratios. For comparisons of the large exon of vertebrate PARP4 (e.g. human and rhesus exon 30), we used K-estimator to distinguish high pairwise dN/dS values due to positive selection from the possibility that these sequences are neutrally evolving, but that stochastic fluctuations in small mutation numbers cause apparently large dN/dS ratio differences. For a pair of sequences with a certain number of observed mutations, K-estimator uses Monte Carlo simulations to obtain "bootstrap" estimates of how likely it would be to see high dN/dS values if sequences were neutrally evolving. For example, comparing human and rhesus PARP4 exon 30 (dN/dS ratio of 1.75), there is greater than 95% confidence that a dN/dS ratio of 1.75 represents a significant signature of positive selection. Sliding window analyses were performed on pairs of aligned vertebrate *PARP4* and *PARP14* sequences with a window size of 150 codons and a step size of 50 codons.

### Phylogenetic analysis

To reconstruct the dynamics of *PARP14* and *15* gain and loss, publically available vertebrate genome assemblies and gene prediction datasets were queried for PARP genes using a combination of blast searches [65], pairwise comparisons of genomic sequences using dotter [66] and the sim4cc program [67] that aligns reference cDNA sequences to genomic sequences from other species. We eliminated from further analysis any PARP sequences that contained frameshifts or nonsense mutations, but retained some genes that were missing up to three exons within genome assembly gaps. Protein sequences were aligned using CLUSTALW [68] with manual adjustment and maximum likelihood phylogenetic trees (1000 bootstrap replicates) were constructed using PhyML [69] using the best-fitting evolutionary model (JTT+I+G+F) as determined by Prottest [70]. Trees were displayed using MEGA [71].

### Structural alignment

Macrodomains from PARP9 and 14 were aligned and mapped to the known structure of the first macrodomain of PARP14 complexed with ADP-ribose (PDB code 3Q6Z) [48]. Figures were generated using PyMol [72].

## Supporting Information

Alignment S1 Primate *PARP4* exon 30. Regions in grey were removed from positive selection analyses due to low confidence in the alignment. Residues highlighted in yellow have evolved under positive selection with a posterior probability >0.95 (See Table S4). The bottom line in each block indicates conservation across all species, with asterisks indicating identical residues and colons representing similar residues.
(DOC)

Alignment S2 Bat *PARP4* largest exon. Regions in grey were removed from positive selection analyses due to low confidence in the alignment. Residues highlighted in yellow have evolved under positive selection with a posterior probability >0.95 (See Table S5). The bottom line in each block indicates conservation across all species, with asterisks indicating identical residues and colons representing similar residues.
(DOC)

Alignment S3 Individual human macrodomains. Human macrodomains (corresponding to positions indicated in italics) were aligned. Residues highlighted in yellow have evolved under positive selection with a posterior probability >0.95 (See Tables S6-S8). The bottom line in each block indicates conservation across all species, with asterisks indicating identical residues and colons representing similar residues.
(DOC)

Figure S1 Additional evolutionary analyses on PARP genes. (A) Results of branch-site analyses for episodic positive selection for each PARP gene using Branch-site REL. Lineages displaying a statistically significant signature of episodic positive selection (P-value <0.05) are indicated. Boxed left empty indicate no significant signature of episodic positive selection. PARP genes in bold red are those that emerged from our initial screen as evolving under strong recurrent positive selection. (B) Estimates of the percent of codons evolving under positive selection and the dN/dS ratio of those codons from the M8 model of PAML. Boxes left empty indicate that the gene lacked statistically significant support for recurrent positive selection. (C) Whole gene dN/dS ratios from the M0 model of PAML.
(PDF)

Figure S2 Lineage specific evolution of the rapidly evolving exon of *PARP4*. (A) Sequences of the largest exon of *PARP4* in primates (corresponding to human exon 30) were subjected to maximum likelihood analyses in PAML using the free ratio model, which allows the dN/dS ratio to vary across the phylogenetic tree. Major primate delineations are indicated (Hominoids - black, Old World monkeys - green, New World monkeys - blue). Values indicated on the phylogenetic tree are dN/dS (decimal values) or nonsynonymous:synonymous ratios (values in parentheses) for each branch calculated using PAML. Branches shown in thick red lines indicate statistically significant signatures of positive selection along that lineage as determined by Branch-site REL. (B) An expanded view of the largest bat *PARP4* exon (corresponding to human *PARP4* exon 30). Above the line are the six codons evolving under recurrent positive selection in an analysis of 10 bat species (red triangles indicate posterior probability > 0.95, as in Figure 2). Nine amino acid residues that are strictly conserved between all 25 primates and bats sampled are marked in green below the line. (C) Expanded view of the sequence alignment of 10 bat species (megabats – purple, microbats – black). Values indicated on the phylogenetic tree to the left and bold red branches as in part A.
(PDF)

Figure S3 No other primate macrodomain-containing or ADP-ribosylhydrolase proteins are evolving under positive selection. (A) Schematic domain structures of human macrodomain-containing proteins as well as non-macrodomain containing proteins that have been shown to catalyze ADP-ribosylhydrolase activity (not to scale). Numbers to the bottom right of the protein schematic indicate the total length, in amino acids, of each protein. CRAL-TRIO: cellular retinaldehyde-binding protein-triple functional domain protein. (B) Results of maximum likelihood tests for positive selection as in Figure 1.
(PDF)

Figure S4 Lineage specific evolution of the macro-PARP genes. (A–C) Same as Figure S2A, except using sequences from *PARP9* (A), *PARP14* (B), or *PARP15* (C) genes.
(PDF)

Figure S5 Alignment of human and viral macrodomains. Representative viral macrodomains were aligned to individual macrodomains from human macro-PARPs. Three motifs noted by Jankevicius et al. [16] to be critical for coordination of ADPr by macrodomains are boxed. Red residues match the conserved consensus sequences at these positions. Asterisks indicate the residues most important for catalytic removal of ADPr from a substrate protein. The lack of conservation of these residues in many of the macro-PARP domains suggests they may be able to bind, but not catalyze removal of, ADPr.
(PDF)

Figure S6 Phylogenetic tree of intact vertebrate *PARP14* and *PARP15* genes. *PARP14* and *PARP15* genes from vertebrates were aligned as described in Materials and Methods being careful to include only open-reading frames that were uninterrupted by frameshifts or stop codons. Some otherwise intact genes are omitted from the tree because one or more exons are missing due to assembly gaps. The maximum likelihood tree generated from these sequences is shown with bootstrap values indicated. We note that the position of marsupial *PARP14*-like sequences in this tree implies that the partial *PARP14* duplication that gave rise to *PARP15* occurred before the divergence of marsupials and placental mammals; however, we have seen no evidence for the presence of *PARP15* in marsupial genomes, and the use of alternate phylogeny inference parameters places the *PARP14-15* duplication after marsupial-placental mammal divergence. We therefore conservatively suggest that the *PARP14-15* duplication occurred after marsupial-placental mammal divergence, rather than the less parsimonious possibility that the duplication occurred earlier and that *PARP15* was subsequently lost in the marsupial ancestor.
(PDF)

Figure S7 Sliding window analyses of recently duplicated *PARP14* paralogs. A sliding window dN/dS analysis (window size 150 codons, step size 50 codons) of *PARP14* paralogs from microbat (A) and bushbaby (B) with the PARP14 domain structure indicated below. The grey horizontal line marks a dN/dS value of 1, indicating neutral evolution. The bushbaby genes fall in a gapped region of the genome assembly, resulting in a central region of the alignment being unreliable and therefore excluded from analysis.
(PDF)

Table S1 Source of primate sequences. ^1^Public genome sequences were used when available. ^2^Cell lines for primates were obtained from the indicated sources and used for amplification of the indicated PARP gene as described in Materials and Methods.
(DOC)

Table S2 Primers used for primate sequence analysis. ^1^List of primers used for amplification of PARP genes from primate DNA (*PARP4*) or RNA (*PARP9*, *14* and *15*). ^2^ *PARP9* and *14* were amplified in sections with the corresponding primer pairs indicated.
(DOC)

Table S3 Whole region dN/dS estimates for *PARP4* largest exon. ^1^The largest exon of indicated *PARP4* genes (corresponding to human exon 30) were aligned and subjected to analysis by K-estimator. ^2^Estimated Ka and Ks values as determined by K-estimator. 95% confidence interval values are shown in italics. ^3^The dN/dS ratio as determined by dividing the estimated Ka value by the estimated Ks value. ^4^Percent confidence that the observed dN/dS ratio is >1 (indicative of the region evolving under positive selection) as calculated by K-estimator.
(DOC)

Table S4 Residues evolving under positive selection in exon 30 of primate *PARP4*. ^1^The largest *PARP4* exon in the human reference sequence (NP_006428.2) is exon 30. Other primate species may have different exon numbering. Residue numbering corresponds to this reference sequence. ^2^Residues with recurrent signatures of positive selection with a posterior probability greater than 0.95 were identified using a Bayes Empirical Bayes (BEB) analysis in PAML from the F3×4 codon frequency model. ^3^Estimated dN/dS ratios from PAML. ^4^Estimated errors for the indicated dN/dS ratio.
(DOC)

Table S5 Residues evolving under positive selection in exon 30 of bat *PARP4*. ^1^The largest *PARP4* exon in the microbat (*Myotis lucifugus*) reference sequence (XP_006085545.1) is exon 30. Other bat species may have different exon numbering. Residue numbering corresponds to this reference sequence. ^2^Residues with recurrent signatures of positive selection with a posterior probability greater than 0.95 were identified using a Bayes Empirical Bayes (BEB) analysis in PAML from the F3×4 codon frequency model. ^3^Estimated dN/dS ratios from PAML. ^4^Estimated errors for the indicated dN/dS ratio.
(DOC)

Table S6 Residues evolving under positive selection in primate *PARP9*. ^1^Residue numbering corresponds to the human reference sequence (XP_005247877.1). ^2^Known protein domains are indicated. ^3^Residues with recurrent signatures of positive selection with a posterior probability greater than 0.95 were identified using a Bayes Empirical Bayes (BEB) analysis in PAML from the F3×4 codon frequency model. ^4^Estimated dN/dS ratios from PAML. ^5^Estimated errors for the indicated dN/dS ratio.
(DOC)

Table S7 Residues evolving under positive selection in primate *PARP14*. ^1^Residue numbering corresponds to the human reference sequence (NP_060024.2). ^2^Known protein domains are indicated. ^3^Residues with recurrent signatures of positive selection with a posterior probability greater than 0.95 were identified using a Bayes Empirical Bayes (BEB) analysis in PAML from the F3×4 codon frequency model. ^4^Estimated dN/dS ratios from PAML. ^5^Estimated errors for the indicated dN/dS ratio.
(DOC)

Table S8 Residues evolving under positive selection in primate *PARP15*. ^1^Residue numbering corresponds to the human reference sequence (NP_001106995.1). ^2^Known protein domains are indicated. ^3^Residues with recurrent signatures of positive selection with a posterior probability greater than 0.95 were identified using a Bayes Empirical Bayes (BEB) analysis in PAML from the F3×4 codon frequency model. ^4^Estimated dN/dS ratios from PAML. ^5^Estimated errors for the indicated dN/dS ratio.
(DOC)
