# Supplementary material for: Rapid Evolution of PARP Genes Suggests a Broad Role for ADP-Ribosylation in Host-Virus Conflicts
Source: PLoS Genet. 2014 May 29;10(5):e1004403. doi: 10.1371/journal.pgen.1004403 (PMC4038475; doi:10.1371/journal.pgen.1004403)
Supplement: Alignment S1 — Primate PARP4 exon 30. Regions in grey were removed from positive selection analyses due to low confidence in the alignment. Residues highlighted in yellow have evolved under positive selection with a posterior probability >0.95 (See Table S4). The bottom line in each block indicates conservation across all species, with asterisks indicating identical residues and colons representing similar residues. (DOC) [file pgen.1004403.s001.doc]

**Alignment S1. Primate *PARP4* exon 30.**

Human LLASSEWPELRLSKRKHRKIPFSKRK----MELSQPEVSEDFEEDGLGVLPAFTSNLERG 56

Chimpanzee LLASSEWPELRLSKRKRRKIPFSKRK----MELSQPEVSEDFEEDALGVLPAFTSNLERG 56

Orangutan LLASSEWQKLYSSKPKLR----AKRK----KKLSQPEVSEDFEEDGLGVLPAFTSNLERG 52

White-cheeked-gibbon LLASSEWQELRSSKPKLR----AKRK----KKLSQPEVSEDFEEDGLGVLPAFTSNLERG 52

Baboon LLASSDWQEVRSSKR-------AKRK----MTLSQPKVFEDVEEDGLDVLPAFTSSLKCE 49

Rhesus LLASSEWQEVRSSKR-------AKRK----MKLSQPKVFEDVEEDGLDVLSAFTSSLKCE 49

Patas LLASSEWQEVRSSKR-------AKRK----MKLSQPKVFEDVEEDGLDVLPAFTSSLKCE 49

Francois-leaf-monkey LLASSEWQEVRSSKRKLR----AKRR----KKLSQPEVFEDVEEDGLGVLPAFTSSLKCG 52

Douc-langur LLASSEWQEVRSSKR-------AKRK----MKLSQPKVFEDFEEDGLDVLPAFTSSLKCE 49

Colobus LLASSEWQEVRSSKRKLR----AKKKKLSQKKLSQPEVFEDVEEDGLGVLPAFTPSLKCE 56

Marmoset LLASSEWQELFSFKRKKCS---SKRK----MKLSKSQVSEDFEEDGLVLLQGFTSNVEHG 53

Squirrel-monkey LLASSEGQDVFSCKRKKRS---SKKK----MKLSKSQVSEDFEEDGLAVPEGFTSNVERG 53 Howler-monkey LLASSEWEELFSPKLKKRS---RKRK----TKLSKSLVSEDLEEDGLAVLQDFTSNVERG 53

Saki LSASSEWQELFSFKKKKCS---AKRK----MKLSKSQVSEDFEEDGLAVLQGFTSRVERG 53

* ***: .: * *:: **:. * **.***.* : **. ::

Human GVEKLLDLSWTESCKPTATEPLFKKVSPWETSTSSFFPILAPAVGSYLPPTARAHSPASL 116

Chimpanzee GVEKLLDLSWTESCKPTATEPLFKKVSPWETSTSSFFPILAPAVGSYLPPTARAHSPASL 116

Orangutan GVEKLSDLSWTESCKPTATEPLFKKVSPWETSTSSLFGVSPPVVGSYLRPTAHAPRAASM 112

White-cheeked-gibbon GVEKLLDLSWTESCKPTATESLFKEVSPQKTSTSSFFPILPPAAGACLPPTARAHGPVSL 112

Baboon GVEKLLDLSWTESCKPTATEPLFRKVSPLETSTSSLFHVSAPAFGSSLPPAARSYSPASV 109

Rhesus GVEKLLDLSRTESCKPTATEPLFRKVSPLETSTSSFFRVSAPAFGSSFPLAARSYSPASM 109

Patas GVEKLLDLSQTESCQPTATEPLFRKVSPLETSTSSFFRVSAPAFGSSLPLAALSYSPASM 109

Francois-leaf-monkey SVEKLLDLSWTESCKPTATEPLFRKVSLLEPSTSSLFGVSAPAFGSSLPPAARPHSPAPM 112

Douc-langur GVEKLLDLSQTESCKPTATEPLFRKVSLLETSTSSLFRVSAPAFGSSLPPAARSYSPASM 109

Colobus SVEKLLDLSWTESCKPTATEPLFRKVSLLETSTSSLFGVSPPAFGSSLPPAARPHSPASM 116

Marmoset HEEKLLDLSWTQSFKPAATEPLFKKVSSPETSTSSLFGFSAPASGSSFFQAAHSHSPASM 113

Squirrel-monkey DEEKILDLSWTQSFKAAATEPLFKKVGSLETSPSSLFGSSAPASGSSFFQAAGSPSPASM 113 Howler-monkey HEEKLLDLSWTQSFKPAATESLFKKVSSPETSTSGLFDSSAPASGSSIFQAARSHSRASM 113

Saki HEEKLLDLSWTQSFKPAATERLFKQVNSMETPTSSLFGFSAPASGSSLFQAARSHSPASV 113

**: *** *:* :.:*** **::*. :...*.:* .*. *: : :* . ..:

Human SFASYRQVASFGSAAPPRQFDASQFSQGPVPGTCADWIPQSASCPTGPPQNPPSSP---- 172

Chimpanzee SFASYRQVASFGSAAPPRQFDASQFSQGPVPGSCADWIPQSASCPTGPPQNPPSAP---- 172

Orangutan PFASYCQAASFGSAAPPRQFDAPQFSQSPAPGSCADWIPQSASCPTGPPQNLSFAP---- 168

White-cheeked-gibbon PFASYGQIASFGSAAPPRQFDASQFSQGPAPGSCADWIPQSASYPTGPPQNLPFPP---- 168

Baboon PFASHGQGAGFGFAPPPRQLAASLFSQGPVPGTCADWIPQSASHPTGPLQKVPCVP---- 165

Rhesus PFASHGQGAGFGFAPPPRQLAASLFSQGPVPGSRADWIPQSASHPTGPLQKVPYVP---- 165

Patas PFASHGQGAGFGFAPPPRELAASLFSQGPVPGSCADWIPQSASHPTGPLQNVPCVP---- 165

Francois-leaf-monkey LFASHGQIAGFGFAPPPRQLDASQFSQGPVPGSCADWIPQSASCPTGPPQNLPCVP---- 168

Douc-langur PFASHGQGAGFGFAPPPRQLAASLFSQGPVPGSCADWIPLLGSHPTGPLQNVPCVP---- 165

Colobus LFASHGQIADLGFSPPPRQLDASQFSQGPVPGSCADWIPQSASCPTGPPQNLPCVP---- 172

Marmoset PLASFPQLPSLGFAAPPRQFDVSEFRKVPVPCSGADWISQSLSCPIEPPQNPPFEP---- 169

Squirrel-monkey PLASFPQLPSLGFAAPPRQFDASDFRNGPVPRRGAARIPQLLSCPIGPPQNPPLPP---- 169

Howler-monkey PLASFPQLPRLGSAAPPRQLDASEFRKGPVPRGGADWISQSLSCPIGPPQNPPFPPVEAT 173

Saki PLASFPQLPSLGFAAPPRRFDASEFRKVPVPR--ADWIPQSLPCPAGAPQNPPLPP---- 167

:**. * . :* :.***.: .. * : *.* * *. . * . *: . *

Human --YCGIVFSGSSLSSAQSAP--LQHPGGFTTRPSAGTFPELDSPQLHFSLPTDPDPIRGF 228

Chimpanzee --YCGIVFSGSSLSSAQSAP--PQPAGGHMTRPSAGTFPELDSPQLHFSVPTDPDPIRGF 228

Orangutan --FCGIAFSGSSLSPTQLPP--PQFPGGCTTRPSAGTSPELDSPQLLFSLPTDPDPIRGF 224

White-cheeked-gibbon --CCGGVFSGSSLSSARSAP--LQPPGGRTTRPSAGTFPELDSPQLHFSLPTDPDPIRGF 224

Baboon --FCGFPFSEGSLSSTQPAP--PPLPGGYTPRPSAGTFPELDSPQLHFSLPTDPDPIRGF 221

Rhesus --FCGFPFSEGSLSSTQPAP--PPLPGGYTPRPSAGTFPELDSPQPHFSLPTDPDPIRGF 221

Patas --FCGFPFSEGSLSSTQPAP--PPLPGGYTPRPSAGTFPELDSPQLRFSLPTDPDPIRGF 221

Francois-leaf-monkey --FCGFAFSEGSLSSTQPAPQPPPLPGGYTPRPSAGTFPALDSPQLHFSLPTDPDPIRGF 226

Douc-langur --FCGFPFSEGSLSSTQPAP--PPLPGGYTPRPSAGTFPELDSPQLHFSLPTDPDPIRGF 221

Colobus --FCGFAFSEGSLNSTQPAPQPPPLPGRYTHRPSAGTFPALDSPQLHCSLPTDPDPIRGF 230

Marmoset --LCGFAYRRSSLLSTQSVP--PEPPGGFMTRPPAGTFPELDSPQVHSSLPTDPDPIKGF 225

Squirrel-monkey --LCGSAFGG-SLFSTQSAP--PKPPGRPMSRPLAGTFPKLHSPQLHSSLPADPDPIKGF 224

Howler-monkey FSLCGFASRGSSLLSTPPAP--PKPPGGVTTRPPAGIFPELDSPQLHSSLPTDPDPIKGF 231

Saki --LCGSASGGSSLLSKRFAP--PKPPGGLMTRPPAGTFPELDSPQLHFSLPTDSDPIKGF 223

** ** . * .* ** ** * *.*** *:*:*.***:**

Human GSYHPSASSPFHFQPSAASLTANLRLPMASALPEALCSQSRTTPVDLCLLEESVGSLEGS 288

Chimpanzee GSYHPSASSPFHFQPSAASLTANLRLPMASALPEALCSQSRTTPLDLCLLEESVGSLEGS 288

Orangutan GSYHLSASSPFHFQPSAASLTANLRLPIASALPEALCSQSQTTPVDLCLLQESVGSLEGS 284

White-cheeked-gibbon GSYHPSASSPFHFQPSTASLTVNLGLPIASALPEPLYSQSQTTPVDLCLLEESVGSLEGS 284

Baboon GSYHPSVSSPFHFQSSAASLTANRRVPIAPALPKTLCSQSWTTPVDLCLLEESVGSLEGS 281

Rhesus GSYHPSVSSPFHFQPSAASLTANLRVPIAPALPKTLCSQSWTTPVDLCLLEESVGSLEGS 281

Patas GSYHPSVSSPFHFQPSAASLTANRRVPIAPALPKTLCSQSWTTPVDICLLEESVGSLEGS 281

Francois-leaf-monkey GSYHPSVSSPFHFQPSAASLTASRRVPIAPALPKTLCSQSWTTPVDLCLLEESGGSLEGS 286

Douc-langur GSYHPSVSSPFHFQPSAASLTANRRVPIAPALPKTLCSQSWTTPVDLCLLEESVDSLEGS 281

Colobus GSYHPSVASPFHFQPSAASLTASRRVPIAPALPKTLCSQSWT-PVDLCLLEESVGSLEGS 289

Marmoset GSYQPSVSSPFHLQPSPASLSTNNGLPVVL--PQALCPLSL---ADLSVQDKSVGSLEGS 280

Squirrel-monkey GSYQPSASSPFHFQPSPASFSTNVGLPVVL---EPLCRRPS---ADLSVQEKSVGSPEGS 278

Howler-monkey GSYQPSVSSPFHFQPSPASLSTKDRLPVVVP--QAVCSLSS---ADLSVQDKSVGSLEES 286

Saki GSYKPCASSPFHFQPSPASLSTTDGLPVVSP--QALCSLSS---ADLSVQDKSVGSLKGS 278

***: ..:****:*.*.**::.. :*:. :.: . *:.: ::* .* : *

Human RCPVFAFQS-SDTESDELSEVLQDSCFLQIKCDTKDDSILCFLEVKEEDEIVCIQHWQDA 347

Chimpanzee RCPVFAFQS-SDTESDELSEVLQDSCFLQIKCDTKDDSILCFLEVKEEDEIVCTQHWQDA 347

Orangutan RCPVFVFRS-SDTESDELSEVLQDSRILQIKCDTKDDSIPCFLEVIEEDEIVCTQHWQDA 343

White-cheeked-gibbon QCPDFGFQSCSETESDELSEVLQDSCFLQIKYDTKDDSILRFLEVIEEDEIVCTQHWQDA 344

Baboon QLPGFGFQS-SETESHELSEVLLDSCFLQIKCDTADDSTPCFPEVIEEDEIVCTQHWQDA 340

Rhesus HLPGFGFQS-SETESHELSEVLLDSCFLQIKYDTADDSIPCFPEVIEEDEIVCTQHWQDA 340

Patas QLPGFGFQS-FETESHELSEVLPDSCFLQIKCDTADDSILCFPEVIEEDEIVCTQHWQGA 340

Francois-leaf-monkey QCPDFGFQS-SETESPELSEVLLDSCFLQIKCDTADDSIPCFPEVIEEDEIVCTQHWQDA 345

Douc-langur QLPGFGFQS-FETESHELSEVLLDSCFLQIKCDTADDSIPCFPEVIEEDEIVCTQHWQDA 340

Colobus QCPDFVFQS-SETESHELSEVLLDSCFLQIKCDTADDGIPCFPEVIEEDEIVCTQHWQDA 348

Marmoset QWLAFDIQS-SETESVELSEVPSNCGYLQMYSGSIVDSTPCLLEVIQEDDIVCTQCWLNA 339

Squirrel-monkey QRLDFVFQT-SETESVELSEVPRNCGYLQLHGDTTVDSTPCLLEVIEEDEVVCIQRWLDA 337

Howler-monkey EWLDFAFHS-SETESVELSEVPRVCGYLQTHSDTIVDSAPCFLKVIEEDEIVCIQRWLDA 345

Saki QWLDFGIQS-AETESVELSEVPRYCGYLQVHSDTIVDSTPCLLEVIEEDEKVCTQRWLDA 337

. * ::: :*** ***** . ** .: *. : :* :**: ** * * .*
