# Supplementary material for: Rapid Evolution of PARP Genes Suggests a Broad Role for ADP-Ribosylation in Host-Virus Conflicts
Source: PLoS Genet. 2014 May 29;10(5):e1004403. doi: 10.1371/journal.pgen.1004403 (PMC4038475; doi:10.1371/journal.pgen.1004403)
Supplement: Alignment S2 — Bat PARP4 largest exon. Regions in grey were removed from positive selection analyses due to low confidence in the alignment. Residues highlighted in yellow have evolved under positive selection with a posterior probability >0.95 (See Table S5). The bottom line in each block indicates conservation across all species, with asterisks indicating identical residues and colons representing similar residues. (DOC) [file pgen.1004403.s002.doc]

**Alignment S2. Bat *PARP4* exon 30.**

Pteropus-vampyrus WRGEFH---LSKRKQKPSTVEVFGVLEGLSLASKAPEKHFRSGWKCTHAE 47

Pteropus-alecto WHGEFH---LSKRKQKPSTVEVFGVLEGLSLASKAPEKHFRSGWKCTHAE 47

Eidolon-helvum LHGEFHPKHLSKRKQKPSTVEVFGDLEGLSLASKAPEQPLKFGWKDAHAE 50

Rhinolophus-ferrumequinum WHEELQCKRLTKRKLKSPRVEVFEDLEVPGLGSQQPEESLTLHFERRDVE 50

Eptesicus-fuscus WHEELQLERLTKKKCKSSKLEVFEGSEMLSLGSHVPEKPLIHNFECRDAE 50

Myotis-davidii WDEELQLECLTKIKCKSSKLEVFKGFGECSLDSHIPEKPLTQNFECRDVE 50

Myotis-lucifugus WDEELQLECLTKKKYKSSKLEVFKGFGECSLGFHIPEKSLTQNFECRDVE 50

Myotis-brandtii WDEELQLECLTKKKCKSSKLEVFKGFGECSLGSHIPEKPLTQNFECRDVE 50

Pteronotus-parnellii WQEESQLKLLTKKKCKSSKQEVFEGFEMCSLGSHLPEIPLKINFEHRDLE 50

Desmodus-rotundus WHEELQLKCLTKKPHKSSKLEVFEGFEVCSLDSHLPEKPLTPNFECRDVE 50

* : *:* *.. *** .* : ** : :: . *

Pteropus-vampyrus TPLDLSQMEPRTCKGAGTPLATAISPQMAVCRSFATPRPAASPAALGSVS 97

Pteropus-alecto TPLDLSQMEPRTCKGAGTPLATAISPQMAVCRSFATPRPAASPAALGSVS 97

Eidolon-helvum MPLDLSQMELCTYKGAGTPLTTAISLPKAGCSPFAAFGLVGSPDALGSLS 100

Rhinolophus-ferrumequinum KPLDLSQMDSLKPKLIEARPAEAISPKMTVPKFFGAPGSARLLAAP-STG 99

Eptesicus-fuscus RPLDLSQIDSFKYTEIKPVFTKASSPTMALSDSFADPGSTSSLAAS-SVG 99

Myotis-davidii RPLDLSQVDSFKHTEIKPHFTKASSPKVALSKSFAAPGSTSSLAAS-SVG 99

Myotis-lucifugus RPLDLSQVDSFKHTEIKPHFTKASSPKVALSKSFAAPGSTSSLAAS-SVG 99

Myotis-brandtii RPLDLSQVDSFKHTEIKPHFTKASSPKVALSKSFAAPGSTSSLAAS-SVG 99

Pteronotus-parnellii RPLDLSLIDSFKHSGIKPAFTKVISPKMALSKSFAASGSASFLAAS-PVG 99

Desmodus-rotundus MPLDLSQIDSFKHSGIKPIFTKVISPKMAVSDSFAAPASTSFLAAS-PIN 99

***** :: . . . : . * : *. . * . .

Pteropus-vampyrus FSHPCPPREPPHSSPFS-FCSAEPSGPATSDLIPEAGCSINVFPEQDSAL 146

Pteropus-alecto FSHPCPPREPPHSSLFS-FCSAELSGPATSDLIPEAGCSINVFPEQDSAL 146

Eidolon-helvum ISRPSSSSAPLHSPLFG-SCGPQLSVPAGSDPGPETGHGTNIFLKQDSVF 149

Rhinolophus-ferrumequinum FSCPRPPNLLPQSSLFGSVARPKQCGPSKNYQGLKTGRRTDTSLVWGSPP 149

Eptesicus-fuscus SAFLYP-----QSSLFGSLASPKQFGPSNNDQGLKTGCSTGIALEWGSPP 144

Myotis-davidii SAFLSP-----RSSLFRSLASSKQFDPPNNEQGLKTDYCPGISLEWGSPP 144

Myotis-lucifugus SAFLYP-----RSSLFGSLASPKQFDPPNNEQGLKTDCCPGVSLQWGSPP 144

Myotis-brandtii SAFLYP-----RSSLFGSLASPKQFDAPNNEQGLKTDCCPGVSLEWGSPP 144

Pteronotus-parnellii STFPCPPNFLPQSPLLGTLACPKPFVPTKNDQGLET----GISLDGGSPP 145

Desmodus-rotundus SASLYPPSLLPPSPLLGTLANSKPFGPAKNDQGLQTGHCTGISLEVGSSP 149

: . *. : . .: .. . :: . .*

Pteropus-vampyrus RGLPPNPPVESFIGPAFAGGLFGSRQCSLPTNYKGLQALGNAGTFLEVDS 196

Pteropus-alecto RGLPPNPPVESFIGPAFAGGLFGSRQCSLPTNYKGLQALGNAGTFLEVDS 196

Eidolon-helvum RWPPPNLSFGSFTGSAFAGGSFGFGQFSLPTTDKGLKAYGKSGMSLEVDS 199

Rhinolophus-ferrumequinum RLP----PFESSPSLALSAASLSSRQFELPKIDDDLDRYSFADISHELDS 195

Eptesicus-fuscus RLPSHSPPFGSFTGSAFSGDLFSCRQSDLPKIYEGPKLCPCTDSSLELDS 194

Myotis-davidii SLPSHNPPFRSFTGSAFSDVLFSYRQSDLPTLNEGPELGPFTDSSLELDS 194

Myotis-lucifugus SLPSHNLPFGSFTGSAFSGVLFSCRQSDLPTMNEGPELCPFTDSSLELDS 194

Myotis-brandtii SLPSHNLPFGSFTGSAFSGVLFSCRQSDLPTLNEGPELCPFTDSSLELDS 194

Pteronotus-parnellii SLPPQKLPFQSFTGSAFPGVLFSSKESYLPKMSEGPQFCPSTDTSLEFNF 195

Desmodus-rotundus SPPPQNPPFGSFSGSAFSGSLFSSRESHLPKMSEGP----ANDSSLDLDF 195

.. * . *:. :. : **. .. . :.:

Pteropus-vampyrus SPQPSPPFSSPSLSPP--FSAADSTLRPLSDFSTFNFMESATPLSTSYMQ 244

Pteropus-alecto SPQPSPPFSSPSLSPP--FSAADSTLLPPSDFSTFNFLESATPLSTSYMQ 244

Eidolon-helvum SPQPSPPFSPPDLPRP--LSATVSSLHPLSDSSTFSFPESAAPLSTSYKQ 247

Rhinolophus-ferrumequinum PPQQNLLLSPMPPPPPSLSLPAEVTFHPLPHSS-LHFPNFPAPLSTSHKQ 244

Eptesicus-fuscus PPQQNPLFYSPRLPPP--GPPGGSSFHLLPGSSPLRFPNSSAPLSTSYKQ 242

Myotis-davidii PPQQNPLFSFPRLPLP--GPAGGSSFHLLPGSSPLYFPTSSAPLSTSYKQ 242

Myotis-lucifugus PPQQNPLFSFPRLPPP--GPTGGSSFHLLPGSSPLYFPNSSAPLSTSYKQ 242

Myotis-brandtii PPQQNPLFSFPRLPPP--GPTGGSSFHLLPGSSPLHFPNSSAPLSTSYKQ 242

Pteronotus-parnellii PPRQNLLFSPPRFPPP--GPTKGCGFQPLLGSSPLHFPNSPGPLSTSYNQ 243

Desmodus-rotundus PLQQNLLFSPPRLPPP--GPTRGSGFQLLPGSS-LQFPNSPVPLSTSYKQ 242

. : . : . * . : * : * . *****: *

Pteropus-vampyrus PAHRLGLASHG----------YGASLSNLPARNAVSRQGLRSAVP----- 279

Pteropus-alecto PAHLLGLGYHG----------YGASPSNLPARNAVSRQGLRSAVP----- 279

Eidolon-helvum PAHPFGLAHHV----------FGASASNLPARNAVSPQGLRSAAP----- 282

Rhinolophus-ferrumequinum PVCLMELDTEARIASSDSPQHVGLAASS-CSDTASDESLFYSSQYAFLCS 293

Eptesicus-fuscus PMRQMGFAHYGAP------PTYEASPSNIAARAASSDSLPHTYLTGL--- 284

Myotis-davidii PMHQIGFAPYGAP------PTCGASPLNISARTASSDSLPHTYLTGL--- 284

Myotis-lucifugus PMHQIGFAPYGP-------PTCGASPLNISARTASSDSLPHTYLTGL--- 283

Myotis-brandtii PMHQIGFAPYGAP------PTCGASPLNISARTASSDSLPHTYLTGL--- 284

Pteronotus-parnellii PIHQIDLALHKAP------PSLGASPSNISVRTAVSDSLPNTCLAAL--- 285

Desmodus-rotundus PIHQIGLVLNEPSPAFRAFPAFGASPSNISVRTALSDSLPDTHLTAL--- 290

* : : . . * . . :

Pteropus-vampyrus ------SSFGTENDETRGFLRITPFKKVAKSHVAGREFTASSQAWENQGP 323

Pteropus-alecto ------RSFGTENDETRGFLRITPFKKVAKSHVAGREFTASSQAWENQGP 323

Eidolon-helvum ------CSFGTENDETQGSLRVKLLKKVAKSPVAGRKLTTSSQAWGNQGF 326

Rhinolophus-ferrumequinum SETENENSFEIESDETLGFLERRLFKGLAKSDRTRRDFPASFRALKDEDD 343

Eptesicus-fuscus ------SSFETESGETPGFLASRLFKKVEKSDITRRDFPASFLVLNDGDD 327

Myotis-davidii ------SSFEIESDETPGFFASSLFKKVEKSDITRRDFPASFPVLNDGDD 327

Myotis-lucifugus ------SSFETESDETPGFFASRLFKKVEKSDITRRDFPASFPVLNDGDD 326

Myotis-brandtii ------SSFETESDETPGFFASRLFKKVENSDIARRDFPASFPVLNDGDD 327

Pteronotus-parnellii ------SSFETKSGEAPGYLASGYFKKLKKCDITRRDFPASLLVLNDEDD 328

Desmodus-rotundus ------SSSETKSDETPGFLASGFFKKVEKSDITKRDFPASFLVLNNEDD 333

* :..*: * : :* : :. : *.:.:* . : .

Pteropus-vampyrus GICQQSQRSSVPWTGLFRLQTE 345

Pteropus-alecto GICQQSQRSSVPWTGLFRLQTE 345

Eidolon-helvum VMCQQSRTSNVPWTELFRLQTE 348

Rhinolophus-ferrumequinum TVCKQSWRNSVPWTDLFSLQTE 365

Eptesicus-fuscus IDCKESWMNFVPGTELFSLQTE 349

Myotis-davidii IDCKESWMNFVPGTELFSLQTE 349

Myotis-lucifugus IDCKESWMNFVPGTELFSLQTE 348

Myotis-brandtii IDCKESWMNFVPGTELFSLQTE 349

Pteronotus-parnellii TDCKESWKNFISVPELFSLQAE 350

Desmodus-rotundus RGCKESWKNFVAVPELLSLQTE 355

*::* . :. . *: **:*
