# Supplementary material for: Rapid Evolution of PARP Genes Suggests a Broad Role for ADP-Ribosylation in Host-Virus Conflicts
Source: PLoS Genet. 2014 May 29;10(5):e1004403. doi: 10.1371/journal.pgen.1004403 (PMC4038475; doi:10.1371/journal.pgen.1004403)
Supplement: Alignment S3 — Individual human macrodomains. Human macrodomains (corresponding to positions indicated in italics) were aligned. Residues highlighted in yellow have evolved under positive selection with a posterior probability >0.95 (See Tables S6-S8). The bottom line in each block indicates conservation across all species, with asterisks indicating identical residues and colons representing similar residues. (DOC) [file pgen.1004403.s003.doc]

**Alignment S3. Individual human macrodomains.**

PARP14 Macro3 *(1227-1354)* -IIFQVASGDITKEEADVIVNSTSNSFNLK-AGVSKAILECAGQNVEREC

PARP15 Macro2 *(304-431)* --TFQVATGDIATEQVDVIVNSTARTFNRK-SGVSRAILEGAGQAVESEC

PARP9 Macro2 *(317-453)* -LTLQIVQGHIEWQTADVIVNSVNPHDITV-GPVAKSILQQAGVEMKSEF

PARP14 Macro2 *(1014-1155)* -LQMLLVKEGVQNAKTDVVVNSVPLDLVLSRGPLSKSLLEKAGPELQEEL

PARP15 Macro1 *(89-243)* -LNLKLISGDVLYIWADVIVNSVPMNLQLGGGPLSRAFLQKAGPMLQKEL

PARP9 Macro1 *(117-295)* RIELSVWKDDLTTHAVDAVVNAANEDLLHG-GGLALALVKAGGFEIQEES

PARP14 Macro1 *(801-977)* GVVLIVQQGDLARLPVDVVVNASNEDLKHY-GGLAAALSKAAGPELQADC

: : : .*.:**: :: :: : .* :: :

PARP14 Macro3 SQ----QAQQRKNDYIITGGGFLRCKNIIHVIG-----GND------VKS

PARP15 Macro2 AV----LAAQPHRDFIITPGGCLKCKIIIHVPG-----GKD------VRK

PARP9 Macro2 LA-TKAKQFQRSQLVLVTKGFNLFCKYIYHVLW-----HSEFPKPQILKH

PARP14 Macro2 DT-VGQGVAVSMGTVLKTSSWNLDCRYVLHVVAPEWRNGST-SSLKIMED

PARP15 Macro1 DD-RRRETEEKVGNIFMTSGCNLDCKAVLHAVAPYWNNGAE-TSWQIMAN

PARP9 Macro1 KQFVARYGKVSAGEIAVTGAGRLPCKQIIHAVGPRWMEWDKQGCTGKLQR

PARP14 Macro1 DQIVKREGRLLPGNATISKAGKLPYHHVIHAVGPRWSGYEAPRCVYLLRR

: . * : : *. :

PARP14 Macro3 SVSSVLQEC--EKKNYSSICLPAIGTGNAKQHPDKVAEAIIDAI---EDF

PARP15 Macro2 TVTSVLEEC--EQRKYTSVSLPAIGTGNAGKNPITVADNIIDAI---VDF

PARP9 Macro2 AMKECLEKC--IEQNITSISFPALGTGNMEIKKETAAEILFDEV---LTF

PARP14 Macro2 IIRECMEIT--ESLSLKSIAFPAIGTGNLGFPKNIFAELIISEV---FKF

PARP15 Macro1 IIKKCLTTV--EVLSFSSITFPMIGTGSLQFPKAVFAKLILSEV---FEY

PARP9 Macro1 AIVSILNYVIYKNTHIKTVAIPALSSGIFQFPLNLCTKTIVETIRVSLQG

PARP14 Macro1 AVQ--LSLCLAEKYKYRSIAIPAISSGVFGFPLGRCVETIVSAIKENFQF

: : :: :* :.:* .. :.. :

PARP14 Macro3 ------------------------------

PARP15 Macro2 ------------------------------

PARP9 Macro2 ------------------------------

PARP14 Macro2 ------------------------------

PARP15 Macro1 ------------------------------

PARP9 Macro1 KPMMSNLKEIHLVSNEDPTVAAFKAASEFI

PARP14 Macro1 KKDGHCLKEIYLVDVSEKTVEAFAEAVKTV
