# Supplementary material for: Rapid Evolution of PARP Genes Suggests a Broad Role for ADP-Ribosylation in Host-Virus Conflicts
Source: PLoS Genet. 2014 May 29;10(5):e1004403. doi: 10.1371/journal.pgen.1004403 (PMC4038475; doi:10.1371/journal.pgen.1004403)
Supplement: Figure S1 — Additional evolutionary analyses on PARP genes. (A) Results of branch-site analyses for episodic positive selection for each PARP gene using Branch-site REL. Lineages displaying a statistically significant signature of episodic positive selection (P-value <0.05) are indicated. Boxed left empty indicate no significant signature of episodic positive selection. PARP genes in bold red are those that emerged from our initial screen as evolving under strong recurrent positive selection. (B) Estimates of the percent of codons evolving under positive selection and the dN/dS ratio of those codons from the M8 model of PAML. Boxes left empty indicate that the gene lacked statistically significant support for recurrent positive selection. (C) Whole gene dN/dS ratios from the M0 model of PAML. (PDF) [file pgen.1004403.s004.pdf]

A

	Branch-site REL
PARP1	
PARP2	
PARP3	
**PARP4**	Marmoset
PARP5a	
PARP5b	
**PARP9**	Marmoset
**PARP14**	
**PARP15**	
PARP7	
PARP12	
**PARP13**	Marmoset Gibbon Rhesus
PARP10	
PARP11	
PARP6	
PARP8	
PARP16	

B

	Positively selected codons	
	%	dN/dS
PARP1		
PARP2		
PARP3		
**PARP4**	5.6	6.0
PARP5a		
PARP5b		
**PARP9**	7.2	6.4
**PARP14**	22	2.7
**PARP15**	8.8	6.9
PARP7		
PARP12		
**PARP13**	20	2.7
PARP10		
PARP11		
PARP6		
PARP8		
PARP16		

C

	Whole gene dN/dS
PARP1	0.13
PARP2	0.20
PARP3	0.28
**PARP4**	0.65
PARP5a	0.04
PARP5b	0.03
**PARP9**	0.85
**PARP14**	0.64
**PARP15**	0.90
PARP7	0.12
PARP12	0.29
**PARP13**	0.71
PARP10	0.32
PARP11	0.12
PARP6	0.01
PARP8	0.07
PARP16	0.09
