# Supplementary figures and images for: Rapid Evolution of PARP Genes Suggests a Broad Role for ADP-Ribosylation in Host-Virus Conflicts
Source: PLoS Genet. 2014 May 29;10(5):e1004403. doi: 10.1371/journal.pgen.1004403 (PMC4038475; doi:10.1371/journal.pgen.1004403)

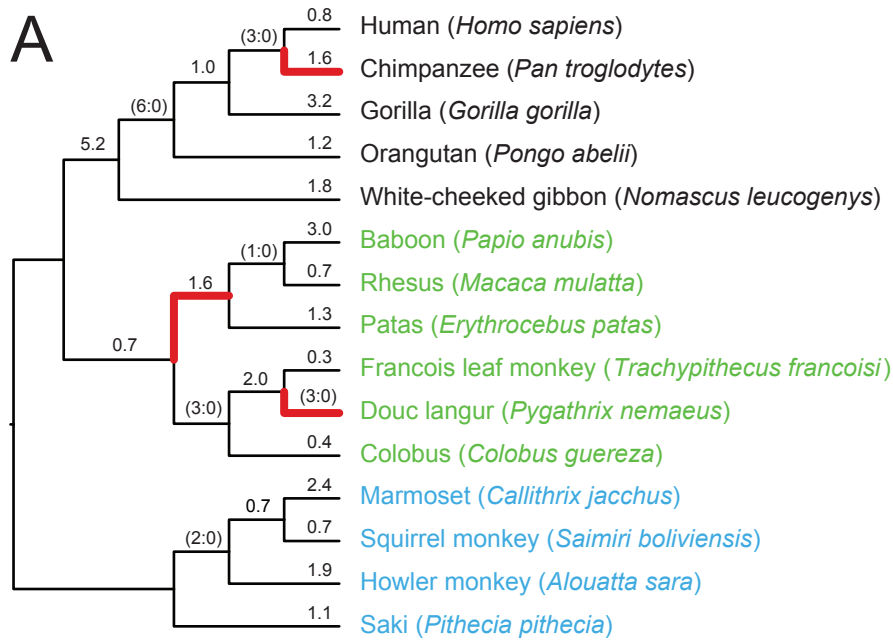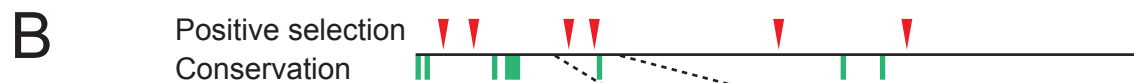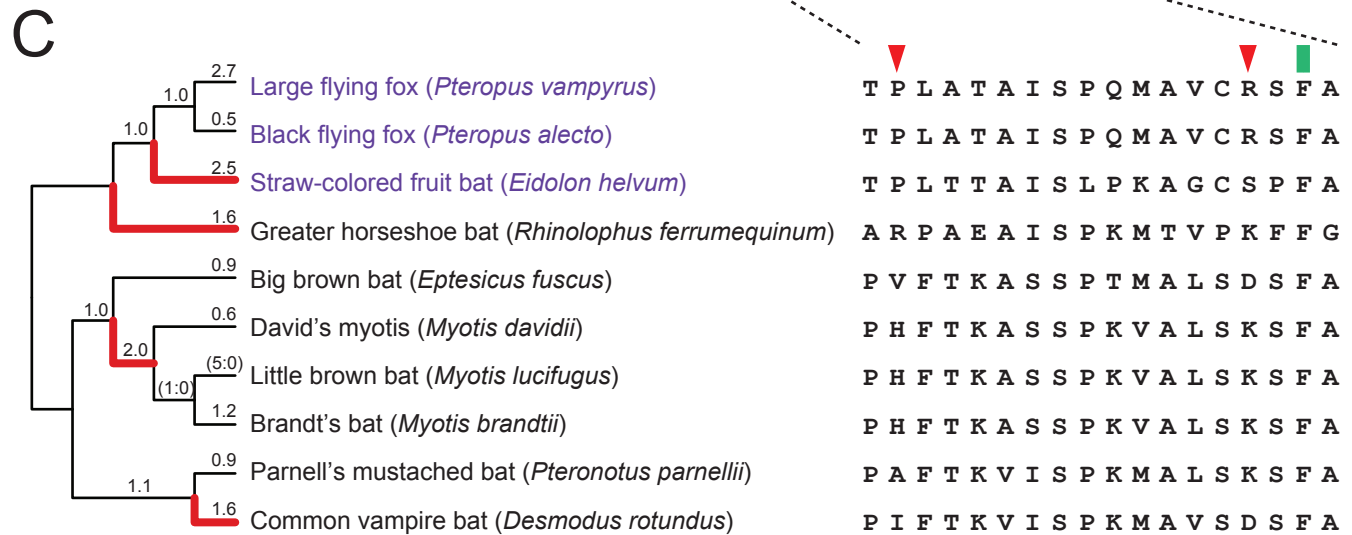

Supplement: Figure S2 — Lineage specific evolution of the rapidly evolving exon of PARP4. (A) Sequences of the largest exon of PARP4 in primates (corresponding to human exon 30) were subjected to maximum likelihood analyses in PAML using the free ratio model, which allows the dN/dS ratio to vary across the phylogenetic tree. Major primate delineations are indicated (Hominoids - black, Old World monkeys - green, New World monkeys - blue). Values indicated on the phylogenetic tree are dN/dS (decimal values) or nonsynonymous:synonymous ratios (values in parentheses) for each branch calculated using PAML. Branches shown in thick red lines indicate statistically significant signatures of positive selection along that lineage as determined by Branch-site REL. (B) An expanded view of the largest bat PARP4 exon (corresponding to human PARP4 exon 30). Above the line are the six codons evolving under recurrent positive selection in an analysis of 10 bat species (red triangles indicate posterior probability > 0.95, as in Figure 2). Nine amino acid residues that are strictly conserved between all 25 primates and bats sampled are marked in green below the line. (C) Expanded view of the sequence alignment of 10 bat species (megabats – purple, microbats – black). Values indicated on the phylogenetic tree to the left and bold red branches as in part A. (PDF) [file pgen.1004403.s005.pdf]

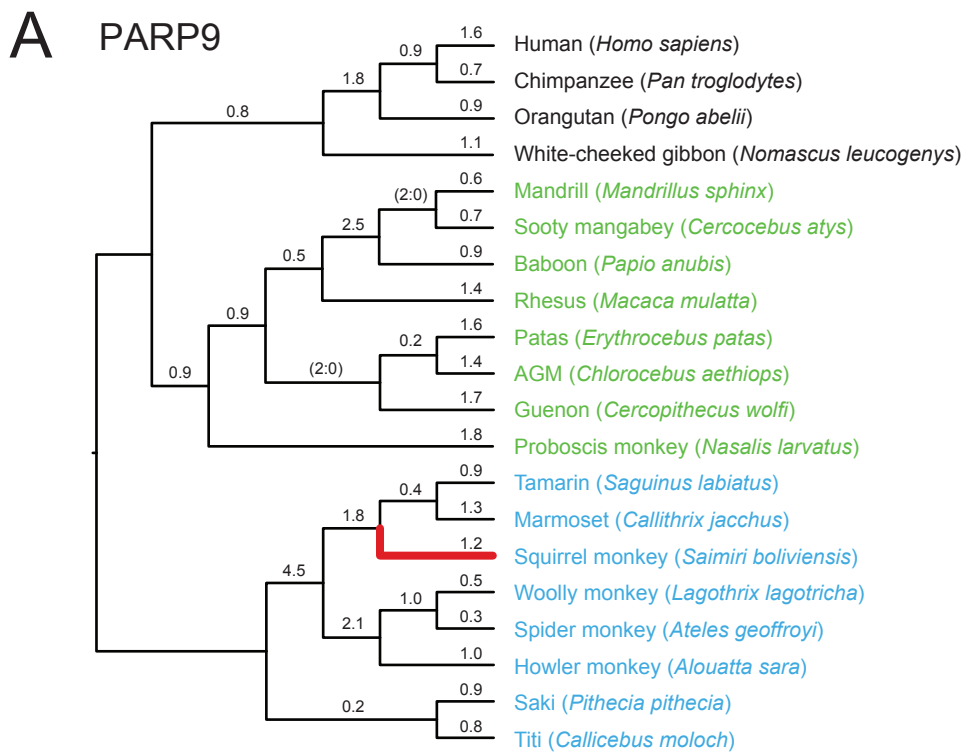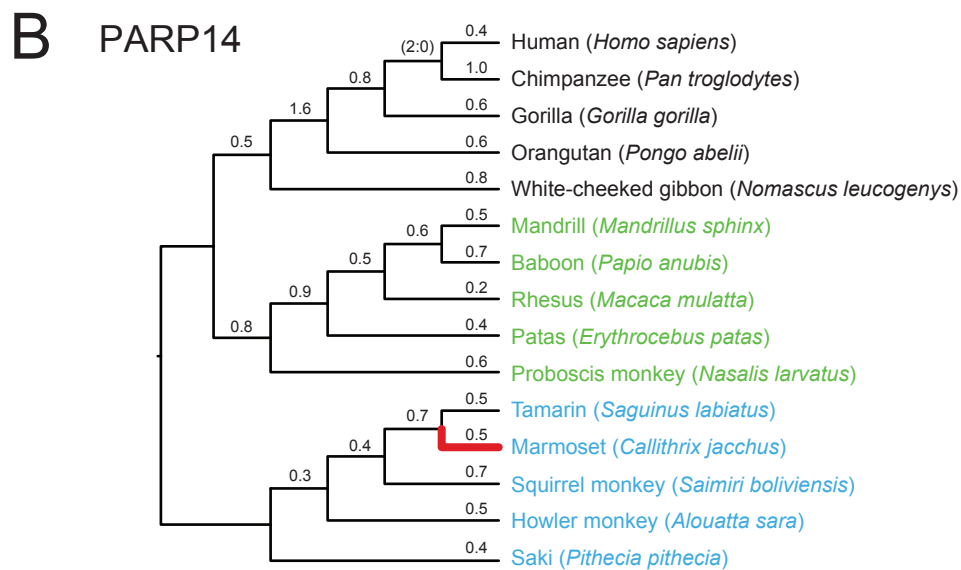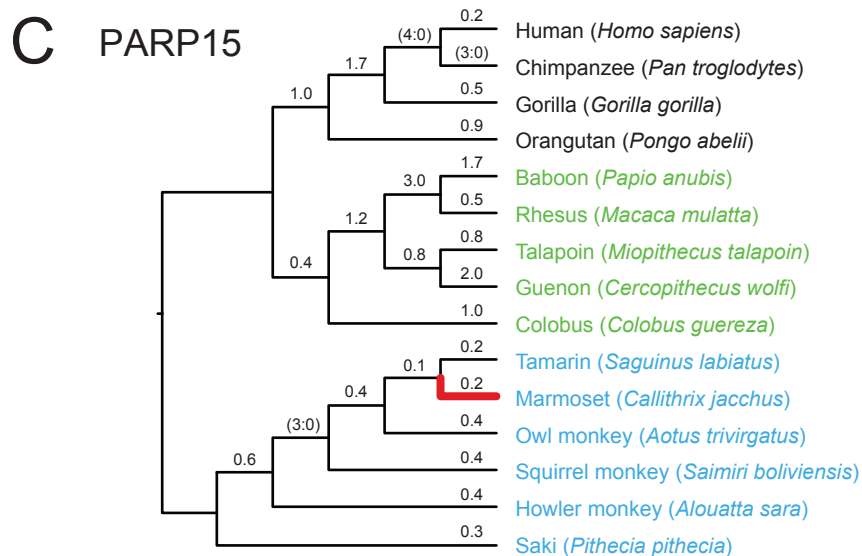

Supplement: Figure S4 — Lineage specific evolution of the macro-PARP genes. (A–C) Same as Figure S2A, except using sequences from PARP9 (A), PARP14 (B), or PARP15 (C) genes. (PDF) [file pgen.1004403.s007.pdf]

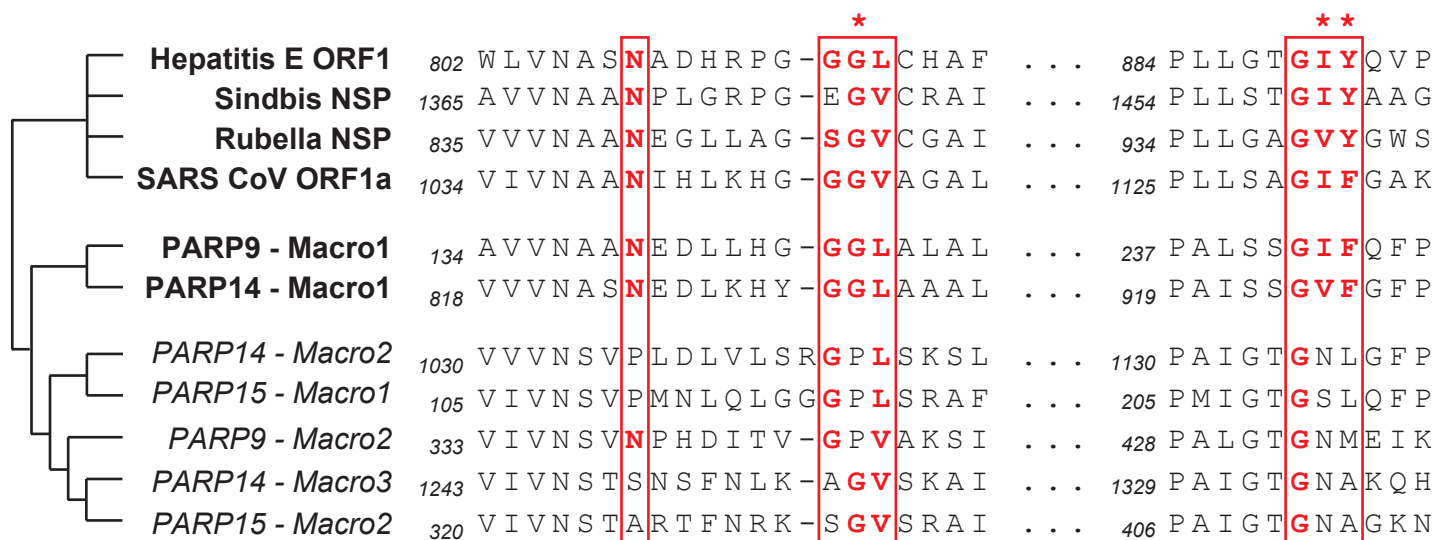

Supplement: Figure S5 — Alignment of human and viral macrodomains. Representative viral macrodomains were aligned to individual macrodomains from human macro-PARPs. Three motifs noted by Jankevicius et al. [16] to be critical for coordination of ADPr by macrodomains are boxed. Red residues match the conserved consensus sequences at these positions. Asterisks indicate the residues most important for catalytic removal of ADPr from a substrate protein. The lack of conservation of these residues in many of the macro-PARP domains suggests they may be able to bind, but not catalyze removal of, ADPr. (PDF) [file pgen.1004403.s008.pdf]

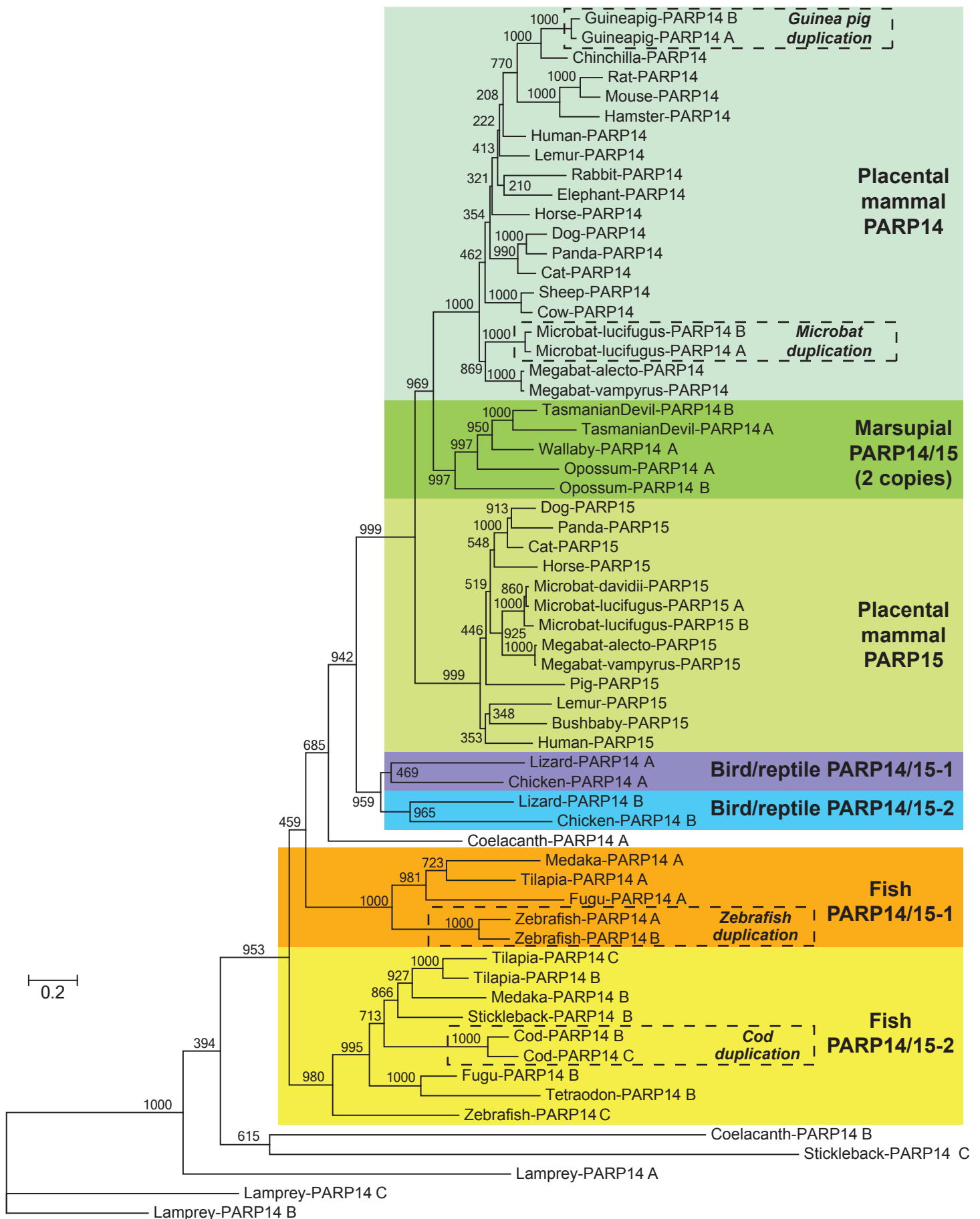

Supplement: Figure S6 — Phylogenetic tree of intact vertebrate PARP14 and PARP15 genes. PARP14 and PARP15 genes from vertebrates were aligned as described in Materials and Methods being careful to include only open-reading frames that were uninterrupted by frameshifts or stop codons. Some otherwise intact genes are omitted from the tree because one or more exons are missing due to assembly gaps. The maximum likelihood tree generated from these sequences is shown with bootstrap values indicated. We note that the position of marsupial PARP14-like sequences in this tree implies that the partial PARP14 duplication that gave rise to PARP15 occurred before the divergence of marsupials and placental mammals; however, we have seen no evidence for the presence of PARP15 in marsupial genomes, and the use of alternate phylogeny inference parameters places the PARP14-15 duplication after marsupial-placental mammal divergence. We therefore conservatively suggest that the PARP14-15 duplication occurred after marsupial-placental mammal divergence, rather than the less parsimonious possibility that the duplication occurred earlier and that PARP15 was subsequently lost in the marsupial ancestor. (PDF) [file pgen.1004403.s009.pdf]

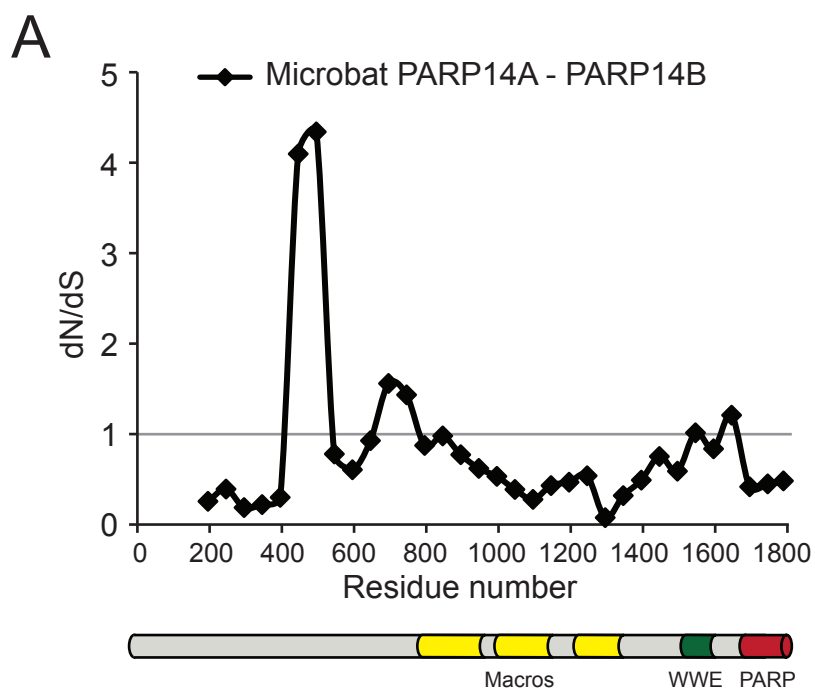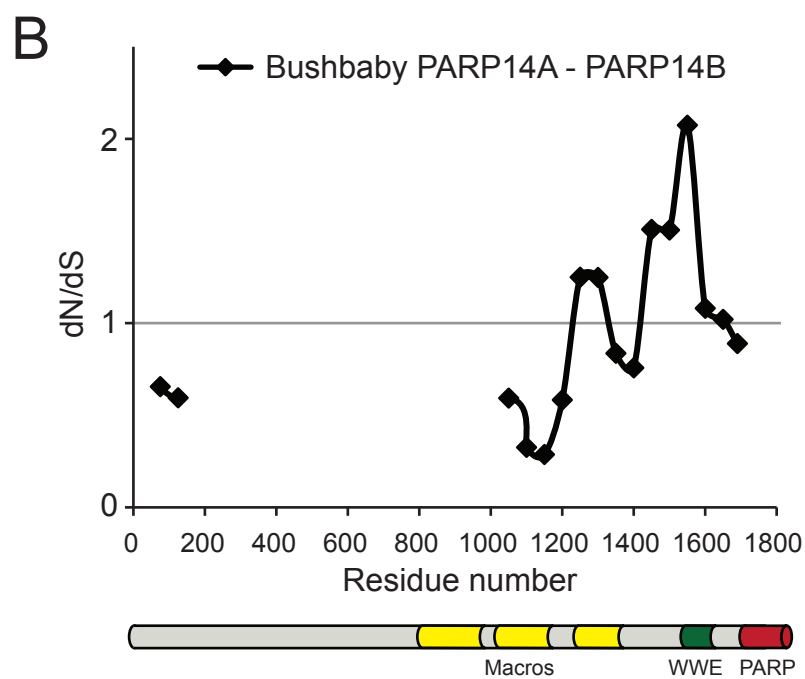

Supplement: Figure S7 — Sliding window analyses of recently duplicated PARP14 paralogs. A sliding window dN/dS analysis (window size 150 codons, step size 50 codons) of PARP14 paralogs from microbat (A) and bushbaby (B) with the PARP14 domain structure indicated below. The grey horizontal line marks a dN/dS value of 1, indicating neutral evolution. The bushbaby genes fall in a gapped region of the genome assembly, resulting in a central region of the alignment being unreliable and therefore excluded from analysis. (PDF) [file pgen.1004403.s010.pdf]
