# Supplementary material for: Rapid Evolution of PARP Genes Suggests a Broad Role for ADP-Ribosylation in Host-Virus Conflicts
Source: PLoS Genet. 2014 May 29;10(5):e1004403. doi: 10.1371/journal.pgen.1004403 (PMC4038475; doi:10.1371/journal.pgen.1004403)
Supplement: Figure S3 — No other primate macrodomain-containing or ADP-ribosylhydrolase proteins are evolving under positive selection. (A) Schematic domain structures of human macrodomain-containing proteins as well as non-macrodomain containing proteins that have been shown to catalyze ADP-ribosylhydrolase activity (not to scale). Numbers to the bottom right of the protein schematic indicate the total length, in amino acids, of each protein. CRAL-TRIO: cellular retinaldehyde-binding protein-triple functional domain protein. (B) Results of maximum likelihood tests for positive selection as in Figure 1. (PDF) [file pgen.1004403.s006.pdf]

A

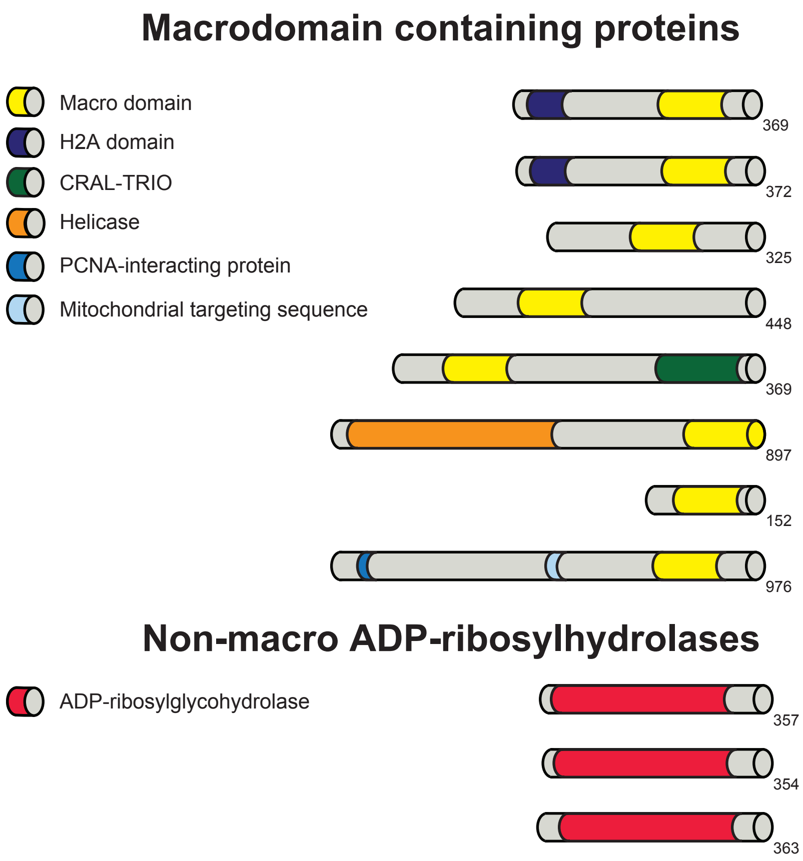

B

Gene name	PAML		PARRIS	
	2 (ln λ)	p-value	LRT	p-value
MacroH2A1	<1.0	>0.4	<0.01	>0.99
MacroH2A2	<1.0	>0.4	<0.01	>0.99
MacroD1	<1.0	>0.4	<0.01	>0.99
MacroD2	<1.0	>0.4	<0.01	>0.99
MacroD2	<1.0	>0.4	<0.01	>0.99
ALC	<1.0	>0.4	<0.01	>0.99
C6ORF130	<1.0	>0.4	<0.01	>0.99
PARG	<1.0	>0.4	<0.01	>0.99
ARH1	<1.0	>0.4	<0.01	>0.99
ARH2	<1.0	>0.4	<0.01	>0.99
ARH3	<1.0	>0.4	<0.01	>0.99
