# Supplementary material for: Rapid Evolution of PARP Genes Suggests a Broad Role for ADP-Ribosylation in Host-Virus Conflicts
Source: PLoS Genet. 2014 May 29;10(5):e1004403. doi: 10.1371/journal.pgen.1004403 (PMC4038475; doi:10.1371/journal.pgen.1004403)
Supplement: Table S1 — Source of primate sequences. 1Public genome sequences were used when available. 2Cell lines for primates were obtained from the indicated sources and used for amplification of the indicated PARP gene as described in Materials and Methods. (DOC) [file pgen.1004403.s011.doc]

**Table S1. Source of primate sequences.**

| **Common name** | **Species Name** | **Source** | **Cell line catalog #** | **PARP sequence** |
| --- | --- | --- | --- | --- |
| Human | *Homo sapiens* | Public genome^1^ |  | 4,9,14,15 |
| Chimpanzee | *Pan troglodytes* | Public genome |  | 4,9,14,15 |
| Gorilla | *Gorilla gorilla* | Public genome |  | 4,9,14,15 |
| Orangutan | *Pongo abelii* | Public genome |  | 4,9,14,15 |
| White-cheeked gibbon | *Nomascus leucogenys* | Public genome |  | 4,9,14 |
| Mandrill | *Mandrillus sphinx* | Coriell^2^ | PR00399 | 9,14 |
| Sooty mangabey | *Cercocebus atys* | Tulane National Primate Research Center | G077 | 9 |
| Baboon | *Papio anubis* | Public genome |  | 4,9,14,15 |
| Rhesus macaque | *Macaca mulatta* | Public genome |  | 4,9,14,15 |
| Patas monkey | *Erythrocebus patas* | Coriell | AG06116 | 4,9,14 |
| Talapoin | *Miopithecus talapoin* | Coriell | PR00716 | 15 |
| African green monkey (AGM) | *Chlorocebus aethiops* | Coriell | PR01190 | 9 |
| Wolf's guenon | *Cercopithecus wolfi* | Coriell | PR01241 | 9,15 |
| Proboscis monkey | *Nasalis larvatus* | Coriell | PR00674 | 9,14 |
| Francois leaf monkey (FLM) | *Trachypithecus francoisi* | Coriell | PR01099 | 4 |
| Douc langur | *Pygathrix nemaeus* | FrozenZoo Project | OR259 | 4 |
| Colobus | *Colobus guereza* | Coriell | PR00980 | 4,15 |
| Tamarin | *Saguinus labiatus* | Coriell | AG05308 | 9,14,15 |
| Marmoset | *Callithrix jacchus* | Public genome |  | 4,9,14,15 |
| Owl monkey | *Aotus trivirgatus* | ATCC | CRL1556 | 15 |
| Squirrel monkey | *Saimiri boliviensis* | Public genome |  | 4,9,14,15 |
| Woolly monkey | *Lagothrix lagotricha* | Coriell | AG05356 | 9 |
| Spider monkey | *Ateles geoffroyi* | Coriell | AG05352 | 9 |
| Howler monkey | *Alouatta sara* | Coriell | PR00708 | 4,9,14,15 |
| Saki monkey | *Pithecia pithecia* | Coriell | PR00239 | 4,9,14,15 |
| Titi monkey | *Callicebus moloch* | Coriell | AG06115 | 9 |
