# Supplementary material for: Rapid Evolution of PARP Genes Suggests a Broad Role for ADP-Ribosylation in Host-Virus Conflicts
Source: PLoS Genet. 2014 May 29;10(5):e1004403. doi: 10.1371/journal.pgen.1004403 (PMC4038475; doi:10.1371/journal.pgen.1004403)
Supplement: Table S2 — Primers used for primate sequence analysis. 1List of primers used for amplification of PARP genes from primate DNA (PARP4) or RNA (PARP9, 14 and 15). 2 PARP9 and 14 were amplified in sections with the corresponding primer pairs indicated. (DOC) [file pgen.1004403.s012.doc]

**Table S2. Primers used for primate sequence analysis.**

| PARP4-exon30-F^1^ | GTCAAGTGCNAGGAAGANTGTTTGC |
| --- | --- |
| PARP4-exon30-R | CACAGATNTAAGGAGAAATAAGACACATACC |
|  |  |
| PARP9-F-1^2^ | CCATATCTGGAAACTACAGTCTATGCTTTGAAG |
| PARP9-R-1 | GCTTGTTGTAGAATTGACTTTGCCACAGGTCC |
| PARP9-F-2 | CCCTACTGTTGCTGCCTTTAAAGCTGC |
| PARP9-R-2 | CCACWTTGCAGAACTGGTRTGGGACTTGC |
| PARP9-F-3 | CAGGARGAAATGGCAAGGAAAAAGGAGC |
| PARP9-R-3 | GCCATCGATGGTCATTATTTGGTTAGTTCACC |
|  |  |
| PARP14-F-1 | CTGGGAGTTTTCCAGGAAACGAAAGCG |
| PARP14-R-1 | CTGAGCCATGGTGTACACCTTTTCYTGG |
| PARP14-F-2 | GTCACTCARCACTTGTGCTTGAAAGGACC |
| PARP14-R-2 | CAGTTTTCACAGCTTCTGCAAAGGCC |
| PARP14-F-3 | CAGCTATTAGTTCTGGAGTCTTTGGCTTTCCC |
| PARP14-R-3 | CTTCAATGGCATCAATTATGGCTTCAGCAACC |
| PARP14-F-4 | CTCATCMATTTGCCTCCCAGCCATTGG |
| PARP14-R-4 | CAAACCTGTTCTTTTCCCACAGATTTGATGTCC |
|  |  |
| PARP15-F | TCCAGAGACGAGTTCAGCAAG |
| PARP15-R | GCTACAAATYGCAGCAATAGAGC |
