# Supplementary material for: Rapid Evolution of PARP Genes Suggests a Broad Role for ADP-Ribosylation in Host-Virus Conflicts
Source: PLoS Genet. 2014 May 29;10(5):e1004403. doi: 10.1371/journal.pgen.1004403 (PMC4038475; doi:10.1371/journal.pgen.1004403)
Supplement: Table S3 — Whole region dN/dS estimates for PARP4 largest exon. 1The largest exon of indicated PARP4 genes (corresponding to human exon 30) were aligned and subjected to analysis by K-estimator. 2Estimated Ka and Ks values as determined by K-estimator. 95% confidence interval values are shown in italics. 3The dN/dS ratio as determined by dividing the estimated Ka value by the estimated Ks value. 4Percent confidence that the observed dN/dS ratio is >1 (indicative of the region evolving under positive selection) as calculated by K-estimator. (DOC) [file pgen.1004403.s013.doc]

**Table S3. Whole region dN/dS estimates for *PARP4* largest exon**

| **Pairwise comparison^1^** | **Ka^2^** | **Ks^2^** | **dN/dS (Ka/Ks)^3^** | **Confidence dN/dS > 1^4^** |
| --- | --- | --- | --- | --- |
| Primates (Human - Rhesus) | 0.127 *(0.100 - 0.156)* | 0.073 *(0.044 - 0.109)* | 1.75 | 97% |
| Carnivores (Dog - Ferret) | 0.236 *(0.196 - 0.279)* | 0.102 *(0.065 - 0.149)* | 2.31 | 99% |
| Birds (Chicken - Turkey) | 0.166 *(0.129 - 0.207)* | 0.081 *(0.043 - 0.122)* | 2.05 | 97% |
| Bats (*M. lucifugus - D. rotundus*) | 0.178 *(0.146 - 0.214)* | 0.115 *(0.074 - 0.159)* | 1.55 | 97% |
| Rodents (Mouse - Rat) | 0.247 *(0.204 - 0.288)* | 0.225 *(0.166 - 0.295)* | 1.10 | 69% |
| Afrotheria (Elephant - Manatee) | 0.103 *(0.082 - 0.130)* | 0.096 *(0.057 - 0.136)* | 1.07 | 60% |
