# Supplementary material for: Rapid Evolution of PARP Genes Suggests a Broad Role for ADP-Ribosylation in Host-Virus Conflicts
Source: PLoS Genet. 2014 May 29;10(5):e1004403. doi: 10.1371/journal.pgen.1004403 (PMC4038475; doi:10.1371/journal.pgen.1004403)
Supplement: Table S4 — Residues evolving under positive selection in exon 30 of primate PARP4. 1The largest PARP4 exon in the human reference sequence (NP_006428.2) is exon 30. Other primate species may have different exon numbering. Residue numbering corresponds to this reference sequence. 2Residues with recurrent signatures of positive selection with a posterior probability greater than 0.95 were identified using a Bayes Empirical Bayes (BEB) analysis in PAML from the F3×4 codon frequency model. 3Estimated dN/dS ratios from PAML. 4Estimated errors for the indicated dN/dS ratio. (DOC) [file pgen.1004403.s014.doc]

**Table S4. Residues evolving under positive selection in exon 30^1^ of primate *PARP4*.**

| **Residue**  **number^1^** | **Posterior probability^2^** | **dN/dS^3^** | **+/-^4^** |
| --- | --- | --- | --- |
| 1238 | 0.987 | 3.427 | 0.401 |
| 1250 | 0.984 | 3.419 | 0.425 |
| 1280 | 0.954 | 3.341 | 0.602 |
| 1289 | 0.993 | 3.443 | 0.348 |
| 1308 | 0.999 | 3.457 | 0.293 |
| 1317 | 0.999 | 3.458 | 0.287 |
| 1332 | 0.957 | 3.348 | 0.589 |
| 1347 | 0.999 | 3.456 | 0.295 |
| 1394 | 0.993 | 3.442 | 0.35 |
| 1407 | 0.964 | 3.366 | 0.555 |
| 1420 | 1 | 3.46 | 0.28 |
| 1474 | 0.955 | 3.342 | 0.601 |
| 1475 | 0.992 | 3.439 | 0.361 |
| 1492 | 0.998 | 3.455 | 0.301 |
| 1513 | 0.999 | 3.458 | 0.288 |
| 1515 | 0.954 | 3.341 | 0.604 |
| 1517 | 0.991 | 3.437 | 0.368 |
| 1533 | 0.975 | 3.396 | 0.486 |
