# Supplementary material for: Rapid Evolution of PARP Genes Suggests a Broad Role for ADP-Ribosylation in Host-Virus Conflicts
Source: PLoS Genet. 2014 May 29;10(5):e1004403. doi: 10.1371/journal.pgen.1004403 (PMC4038475; doi:10.1371/journal.pgen.1004403)
Supplement: Table S5 — Residues evolving under positive selection in exon 30 of bat PARP4. 1The largest PARP4 exon in the microbat (Myotis lucifugus) reference sequence (XP_006085545.1) is exon 30. Other bat species may have different exon numbering. Residue numbering corresponds to this reference sequence. 2Residues with recurrent signatures of positive selection with a posterior probability greater than 0.95 were identified using a Bayes Empirical Bayes (BEB) analysis in PAML from the F3×4 codon frequency model. 3Estimated dN/dS ratios from PAML. 4Estimated errors for the indicated dN/dS ratio. (DOC) [file pgen.1004403.s015.doc]

**Table S5. Residues evolving under positive selection in exon 30^1^ of bat *PARP4*.**

| **Residue**  **number^1^** | **Posterior probability^2^** | **dN/dS^3^** | **+/-^4^** |
| --- | --- | --- | --- |
| 1241 | 0.987 | 2.96 | 0.61 |
| 1268 | 0.981 | 2.949 | 0.628 |
| 1295 | 0.997 | 2.981 | 0.577 |
| 1308 | 0.983 | 2.952 | 0.623 |
| 1390 | 0.954 | 2.891 | 0.702 |
| 1477 | 0.989 | 2.964 | 0.605 |
