# Supplementary material for: Rapid Evolution of PARP Genes Suggests a Broad Role for ADP-Ribosylation in Host-Virus Conflicts
Source: PLoS Genet. 2014 May 29;10(5):e1004403. doi: 10.1371/journal.pgen.1004403 (PMC4038475; doi:10.1371/journal.pgen.1004403)
Supplement: Table S6 — Residues evolving under positive selection in primate PARP9. 1Residue numbering corresponds to the human reference sequence (XP_005247877.1). 2Known protein domains are indicated. 3Residues with recurrent signatures of positive selection with a posterior probability greater than 0.95 were identified using a Bayes Empirical Bayes (BEB) analysis in PAML from the F3×4 codon frequency model. 4Estimated dN/dS ratios from PAML. 5Estimated errors for the indicated dN/dS ratio. (DOC) [file pgen.1004403.s016.doc]

**Table S6. Residues evolving under positive selection in primate *PARP9***.

| **Residue number^1^** | **Domain^2^** | **Posterior probability^3^** | **dN/dS^4^** | **+/-^5^** |
| --- | --- | --- | --- | --- |
| 5 |  | 0.983 | 4.682 | 0.658 |
| 54 |  | 0.969 | 4.625 | 0.804 |
| 63 |  | 0.986 | 4.691 | 0.636 |
| 70 |  | 0.993 | 4.72 | 0.542 |
| 100 |  | 0.987 | 4.698 | 0.616 |
| 118 | Macro1 | 0.973 | 4.642 | 0.764 |
| 168 | Macro1 | 0.957 | 4.577 | 0.911 |
| 172 | Macro1 | 0.964 | 4.604 | 0.849 |
| 179 | Macro1 | 0.992 | 4.715 | 0.559 |
| 204 | Macro1 | 0.961 | 4.595 | 0.875 |
| 214 | Macro1 | 0.953 | 4.56 | 0.935 |
| 218 | Macro1 | 0.994 | 4.724 | 0.529 |
| 227 | Macro1 | 1 | 4.747 | 0.434 |
| 253 | Macro1 | 1 | 4.746 | 0.439 |
| 260 | Macro1 | 1 | 4.747 | 0.435 |
| 265 | Macro1 | 0.993 | 4.722 | 0.536 |
| 294 | Macro1 | 1 | 4.746 | 0.442 |
| 328 | Macro2 | 0.989 | 4.703 | 0.599 |
| 341 | Macro2 | 1 | 4.747 | 0.435 |
| 345 | Macro2 | 1 | 4.746 | 0.441 |
| 362 | Macro2 | 1 | 4.746 | 0.44 |
| 373 | Macro2 | 1 | 4.747 | 0.435 |
| 401 | Macro2 | 1 | 4.747 | 0.437 |
| 403 | Macro2 | 1 | 4.747 | 0.436 |
| 404 | Macro2 | 0.994 | 4.724 | 0.527 |
| 405 | Macro2 | 1 | 4.746 | 0.438 |
| 408 | Macro2 | 0.96 | 4.588 | 0.881 |
| 476 |  | 1 | 4.747 | 0.437 |
| 531 |  | 0.974 | 4.644 | 0.762 |
| 576 |  | 0.983 | 4.682 | 0.658 |
| 587 |  | 0.993 | 4.72 | 0.541 |
| 617 |  | 0.989 | 4.704 | 0.597 |
| 626 |  | 0.961 | 4.593 | 0.88 |
| 646 |  | 0.976 | 4.654 | 0.737 |
| 648 |  | 0.994 | 4.724 | 0.527 |
| 677 |  | 0.962 | 4.597 | 0.864 |
| 845 | PARP | 0.955 | 4.567 | 0.921 |
