# Supplementary material for: Rapid Evolution of PARP Genes Suggests a Broad Role for ADP-Ribosylation in Host-Virus Conflicts
Source: PLoS Genet. 2014 May 29;10(5):e1004403. doi: 10.1371/journal.pgen.1004403 (PMC4038475; doi:10.1371/journal.pgen.1004403)
Supplement: Table S7 — Residues evolving under positive selection in primate PARP14. 1Residue numbering corresponds to the human reference sequence (NP_060024.2). 2Known protein domains are indicated. 3Residues with recurrent signatures of positive selection with a posterior probability greater than 0.95 were identified using a Bayes Empirical Bayes (BEB) analysis in PAML from the F3×4 codon frequency model. 4Estimated dN/dS ratios from PAML. 5Estimated errors for the indicated dN/dS ratio. (DOC) [file pgen.1004403.s017.doc]

**Table S7. Residues evolving under positive selection in primate *PARP14*.**

| **Residue number^1^** | **Domain^2^** | **Posterior probability^3^** | **dN/dS^4^** | **+/-^5^** |
| --- | --- | --- | --- | --- |
| 537 |  | 0.967 | 3.353 | 0.539 |
| 563 |  | 0.973 | 3.371 | 0.492 |
| 611 |  | 0.954 | 3.318 | 0.616 |
| 650 |  | 0.955 | 3.32 | 0.614 |
| 763 |  | 0.989 | 3.413 | 0.366 |
| 888 | Macro1 | 0.986 | 3.407 | 0.386 |
| 894 | Macro1 | 0.966 | 3.351 | 0.545 |
| 903 | Macro1 | 0.999 | 3.44 | 0.251 |
| 906 | Macro1 | 0.99 | 3.417 | 0.353 |
| 947 | Macro1 | 1 | 3.442 | 0.237 |
| 952 | Macro1 | 0.965 | 3.349 | 0.549 |
| 953 | Macro1 | 0.994 | 3.428 | 0.306 |
| 996 |  | 0.989 | 3.413 | 0.366 |
| 1040 | Macro2 | 0.984 | 3.4 | 0.409 |
| 1063 | Macro2 | 0.989 | 3.415 | 0.36 |
| 1272 | Macro3 | 0.98 | 3.389 | 0.443 |
| 1374 |  | 0.958 | 3.328 | 0.596 |
| 1395 |  | 0.97 | 3.361 | 0.521 |
| 1437 |  | 0.964 | 3.346 | 0.556 |
| 1441 |  | 0.992 | 3.421 | 0.336 |
| 1546 | WWE | 0.961 | 3.338 | 0.574 |
| 1574 | WWE | 0.991 | 3.42 | 0.341 |
| 1575 | WWE | 0.998 | 3.437 | 0.263 |
| 1590 | WWE | 0.992 | 3.421 | 0.335 |
| 1599 |  | 0.954 | 3.317 | 0.619 |
| 1625 |  | 0.996 | 3.433 | 0.282 |
| 1675 |  | 0.973 | 3.369 | 0.5 |
| 1707 | PARP | 0.958 | 3.329 | 0.594 |
| 1724 | PARP | 0.974 | 3.373 | 0.487 |
| 1750 | PARP | 0.991 | 3.42 | 0.341 |
| 1778 | PARP | 0.992 | 3.422 | 0.331 |
| 1779 | PARP | 0.985 | 3.404 | 0.397 |
