# Supplementary material for: Rapid Evolution of PARP Genes Suggests a Broad Role for ADP-Ribosylation in Host-Virus Conflicts
Source: PLoS Genet. 2014 May 29;10(5):e1004403. doi: 10.1371/journal.pgen.1004403 (PMC4038475; doi:10.1371/journal.pgen.1004403)
Supplement: Table S8 — Residues evolving under positive selection in primate PARP15. 1Residue numbering corresponds to the human reference sequence (NP_001106995.1). 2Known protein domains are indicated. 3Residues with recurrent signatures of positive selection with a posterior probability greater than 0.95 were identified using a Bayes Empirical Bayes (BEB) analysis in PAML from the F3×4 codon frequency model. 4Estimated dN/dS ratios from PAML. 5Estimated errors for the indicated dN/dS ratio. (DOC) [file pgen.1004403.s018.doc]

**Table S8. Residues evolving under positive selection in primate *PARP15*.**

| **Residue number^1^** | **Domain^2^** | **Posterior probability^3^** | **dN/dS^4^** | **+/-^5^** |
| --- | --- | --- | --- | --- |
| 196 | Macro1 | 0.996 | 4.239 | 1.223 |
| 256 |  | 0.962 | 4.095 | 1.337 |
| 285 |  | 0.998 | 4.246 | 1.216 |
| 286 |  | 0.993 | 4.227 | 1.234 |
| 369 | Macro2 | 0.999 | 4.251 | 1.21 |
| 551 |  | 0.989 | 4.21 | 1.247 |
| 568 | PARP | 0.99 | 4.214 | 1.244 |
